# Protein remote homology detection and structural alignment using deep learning

**DOI:** 10.1038/s41587-023-01917-2

**Published:** 2023-09-07

**Authors:** Tymor Hamamsy, James T. Morton, Robert Blackwell, Daniel Berenberg, Nicholas Carriero, Vladimir Gligorijevic, Charlie E. M. Strauss, Julia Koehler Leman, Kyunghyun Cho, Richard Bonneau

**Affiliations:** 1 Center for Data Science, New York University, New York, NY USA; 2 Center for Computational Biology, Flatiron Institute, Simons Foundation, New York, NY USA; 3 Biostatistics and Bioinformatics Branch, Eunice Kennedy Shriver National Institute of Child Health and Human Development, National Institutes of Health, Bethesda, MD USA; 4 Scientific Computing Core, Flatiron Institute, Simons Foundation, New York, NY USA; 5 Department of Computer Science, Courant Institute of Mathematical Sciences, New York University, New York, NY USA; 6 Prescient Design, New York, NY USA; 7 Bioscience Division, Los Alamos National Laboratory, Los Alamos, NM USA; 8 CIFAR, Toronto, Ontario Canada; 9 Department of Biology, New York University, New York, NY USA

**Keywords:** Machine learning, Sequence annotation, Protein databases, Data mining, Computational models

## Abstract

Exploiting sequence–structure–function relationships in biotechnology requires improved methods for aligning proteins that have low sequence similarity to previously annotated proteins. We develop two deep learning methods to address this gap, TM-Vec and DeepBLAST. TM-Vec allows searching for structure–structure similarities in large sequence databases. It is trained to accurately predict TM-scores as a metric of structural similarity directly from sequence pairs without the need for intermediate computation or solution of structures. Once structurally similar proteins have been identified, DeepBLAST can structurally align proteins using only sequence information by identifying structurally homologous regions between proteins. It outperforms traditional sequence alignment methods and performs similarly to structure-based alignment methods. We show the merits of TM-Vec and DeepBLAST on a variety of datasets, including better identification of remotely homologous proteins compared with state-of-the-art sequence alignment and structure prediction methods.

## Abstract (web summary)

Protein sequence and structural similarities in large databases are rapidly identified using machine learning.

## Main

Detecting protein sequence homology using sequence similarity is the standard approach to identifying evolutionarily conserved functions that are common between proteins^1,2^. During the past 50 years, sequence homology has enabled a wide array of applications, including annotating protein functions^3–7^, predicting protein structure and protein interactions^8–13^, aiding protein design^14^ and modeling evolutionary relationships^1^.

Many standard sequence homology approaches are reliable for proteins that have high sequence similarity (>25%). However, unlike sequence homology, structural homology can be retained across long evolutionary timescales^15^. More than half of all proteins do not have sequence homology in standard sequence databases owing to their distant evolutionary relationships^16^. Recent metagenomics studies have shown that the annotation rate could be boosted up to 70% using structural homology detection^17^. The challenge of remote homology detection is identifying structurally similar proteins that do not necessarily have high sequence similarity. It is widely understood that protein structure–structure alignments offer substantially more structure–function value at longer evolutionary distances that typically elude methods based on protein sequence alignment. Using sequence-alignment-based methods for closely related proteins and structure-alignment-based methods for distantly related proteins could be an ideal hybrid approach offering substantially better sensitivity.

When protein structures are available, structural alignment tools such as TM-align^15^, Dali^18^, FAST^19^ and Mammoth^20^ can provide a measure of structural similarity by aligning protein structures via superposition^15,18,20–22^. Although this approach can provide a measure of structural similarity in low-sequence-similarity scenarios, there are two major limitations. First, protein structures are not available for most proteins. Despite the rapid advances made by AlphaFold2, there remains a large gap between known protein sequences and predicted protein structures^23^. In metagenomics samples alone, 2.4 billion^24^ to 68 billion^25^ unique proteins have been observed, highlighting the small percentage of proteins with known structures. Furthermore, AlphaFold2 has limited utility in the context of predicting structures for proteins with short sequences^26^. Work on structure prediction that uses single or few homologous sequences is ongoing, but most methods exhibit reduced accuracy and take substantial time and memory resources per sequence, limiting scaling to genomic protein databases.

Given the rapid growth of protein structure databases, most existing structural alignment tools are far too computationally intensive to run at scale, requiring brute-force all-versus-all comparisons to query structurally similar proteins. Although there are emerging tools for scalable homology search on structural databases^27^, as well as for embedding proteins for either search or alignment^28,29^ (Table 1), tools that perform explicit structural similarity search and alignment on large protein sequence databases are also needed.

**Table 1:** Inputs and outputs of methods used for benchmarking

	Input data		Output alignment	
Tool	Sequence	Structure	Sequence	Structure
Needleman–Wunsch	*✓*		*✓*	
Smith–Waterman	*✓*		*✓*	
BLAST	*✓*		*✓*	
HMMER	*✓*		*✓*	
Diamond	*✓*		*✓*	
HHBlits	*✓*		*✓*	
MMseq2	*✓*		*✓*	
ProtTucker/EAT	*✓*		*✓*	
FoldSeek		*✓*	*✓*	*✓*
TM-align		*✓*	*✓*	*✓*
Dali		*✓*	*✓*	*✓*
FAST		*✓*	*✓*	*✓*
Mammoth		*✓*	*✓*	*✓*
**TM-Vec** **+** **DeepBLAST**	*✓*		*✓*	*✓*

Our pipeline, consisting of TM-Vec + DeepBLAST, is highlighted.

To enable scalable structurally aware search and alignments on protein sequences, we developed two tools, TM-Vec and DeepBLAST. TM-Vec can compute accurate structural similarity scores; it outputs vector representations of proteins and can be used to construct indexable databases to enable efficient querying of proteins by structural similarity. DeepBLAST can compute structural alignments from pairs of sequences. Building on recent advances in protein language models^30–36^, we developed neural networks that can be fine-tuned on protein structures to (1) predict TM-scores between pairs of proteins using twin neural networks and (2) predict structural alignments between proteins using a differentiable Needleman–Wunsch algorithm.

We showcase the merits of TM-Vec models in the context of CATH^37^ and SWISS-MODEL^38^ to show how our tool can scale with regard to database size while maintaining high precision in identifying structurally similar proteins. Our benchmarks suggest that TM-Vec can extrapolate beyond known fold space, and we contrast TM-Vec with AlphaFold2 (ref. ^10^), OmegaFold^39^ and ESMFold^40^ in a case study where TM-Vec can distinguish between bacteriocin classes more accurately than AlphaFold2, OmegaFold and ESMFold in combination with TM-align^11^. We also showcase the merits of DeepBLAST on different remote homology benchmarks, demonstrating that language model embeddings can capture more of the structural basis for alignment than purely sequence-based alignment. TM-Vec and DeepBLAST are broadly applicable tools that have the potential to enable the structural (and structural-similarity-based) annotation of proteins and their functions in the vast and growing biodiversity contained in protein sequence collections.

## Results

**Fig. 1:**
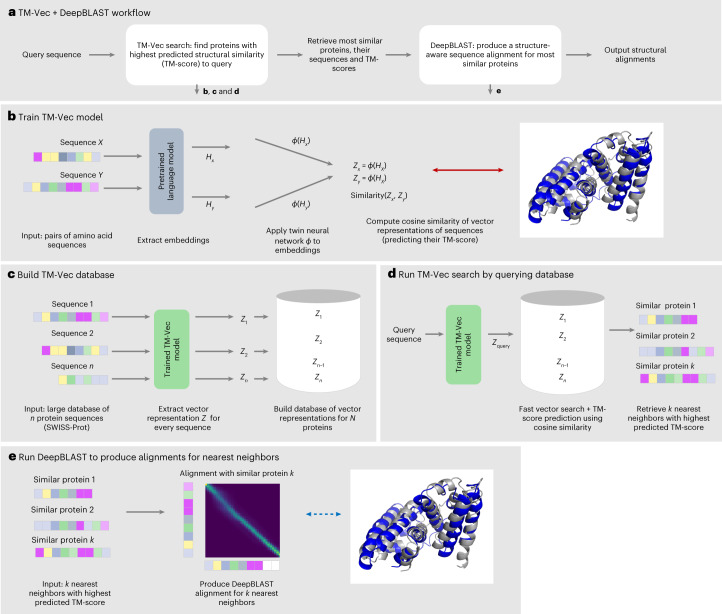
Schematic method overview. **a**, An integrated TM-Vec + DeepBLAST pipeline could consist of two stages: retrieval and alignment. First, TM-Vec takes a query protein sequence and rapidly retrieves proteins that are predicted to have similar structures (TM-scores) to the query. Then, DeepBLAST produces alignments for the proteins with the highest predicted structural similarity. Note that benchmarking was carried out for TM-Vec and DeepBLAST separately. **b**, TM-Vec is trained on pairs of amino acid sequences and their TM-scores. We first input a pair of sequences (domains, chains, proteins) and use a pretrained deep protein language model to extract embeddings for every residue of the sequence. Next, we apply a twin neural network, called *ϕ*, to the embeddings of each sequence and produce a vector representation, **z**, for each sequence. The *ϕ* network is trained on millions of pairs of sequences, and its architecture is detailed in Supplementary Fig. 1. Finally, we compute the cosine similarity of the vector representations, which is our prediction for the TM-score of the pair. **c**, We build a TM-Vec database by encoding large databases of protein sequences using a trained TM-Vec model. As an example, we input the sequences from Swiss-Prot, extract vector representations for every sequence and finally build an indexed database of TM-Vec’s structure-aware vector representations of proteins. **d**, Demonstration of protein structure search using the TM-Vec pipeline. As the indexed database of vector representations has already been built, protein search consists of first encoding the query sequence using the trained TM-Vec model and then performing fast vector search and TM-score prediction using cosine similarity as the search metric. As search results, we return the *k* nearest neighbors with the highest predicted structural similarity (TM-score) to the query sequence. **e**, As a last step, we apply DeepBLAST to produce structural alignments for the *k* nearest neighbors to a query sequence.

Our contributions are twofold: (1) we introduce a framework to perform scalable structure-aware search, TM-Vec, that affords substantial improvements in speed and sensitivity^41^ (Fig. 1 and Supplementary Fig. 1); and (2) we introduce a differentiable sequence alignment algorithm, DeepBLAST, that performs structural alignments (Supplementary Fig. 2).

TM-Vec is a twin neural network model that produces protein vectors that can be efficiently indexed and queried^41,42^ (Fig. 1). To encode structural information in these protein vectors, TM-Vec is trained to approximate TM-scores (as a metric of structural similarity) of pairs of proteins with structures. Once a TM-Vec model has been trained, it can be used to encode large databases of protein sequences, producing structure-aware vector embeddings for these protein sequences. Upon creation of the TM-Vec vector-embedding database, rapid protein structure search is possible by finding the nearest neighbors in the embedding space.

The basis of DeepBLAST is to predict the structural alignments of proteins by training models on proteins with both sequences and structures available. Our alignment strategy uses recent developments in differentiable dynamic programming and protein language models to predict the structural alignments given by TM-align for pairs of protein sequences (Supplementary Fig. 2).

We showcase the ability of DeepBLAST to extract structural alignments from remote homologs on the Malidup^43^ and Malisam^44^ structure databases compared with existing alignment algorithms. Furthermore, we evaluate the ability of TM-Vec to perform remote homology search on the CATH^37^, SWISS-MODEL^38^, Malidup^43^ and Malisam^44^ structure databases. Finally, we showcase the merits of using TM-Vec in tandem with DeepBLAST in the context of the BAGEL bacteriocin database^45^.

### Scalable structural alignment search using neural networks

The challenge of applying our proposed structural alignment algorithm to large-scale protein databases is the demanding runtime requirements. Each DeepBLAST structural alignment takes on the order of milliseconds and scales linearly with database size, making structural alignment searches on large databases impractical. To mitigate this issue, we developed TM-Vec, a model that is designed to efficiently query structurally similar proteins. Our strategy relies on the construction of twin neural networks, whose purpose is to provide per-protein vectors for fast indexing. The cosine distance of these vectors approximates the TM-score between pairs of proteins. This model can then be applied to entire protein databases to create an index over all the protein vectors. The resulting database can be efficiently queried in $O({\log }^{2}n)$ time for *n* proteins^41^, providing sublinear scaling to retrieve structurally similar proteins based on their TM-score.

**Fig. 2:**
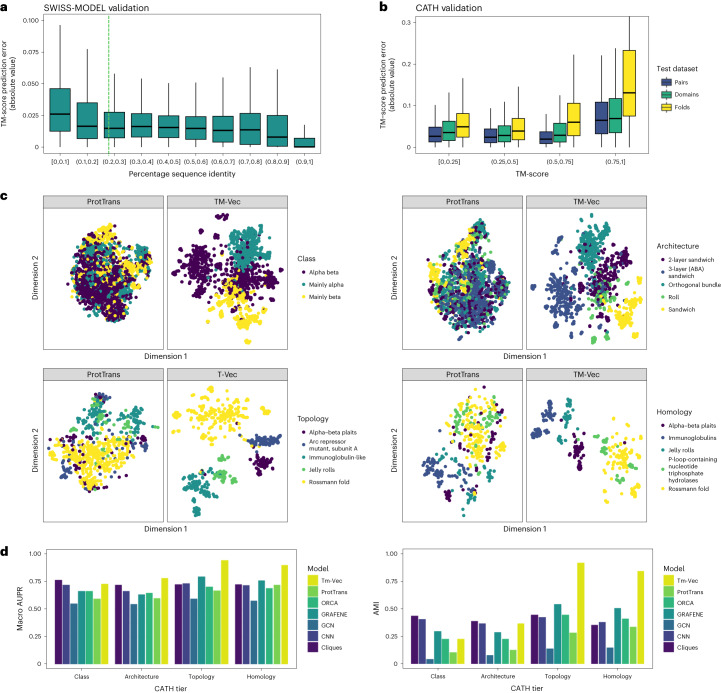
TM-Vec structural similarity prediction. **a**–**d**, Two TM-Vec models were built and benchmarked against protein pairs from SWISS-MODEL and CATHS40. **a**, SWISS-MODEL TM-score prediction errors (absolute value of difference between the known TM-score from running TM-align on structures and the TM-Vec-predicted TM-score) for 1.01 million pairs with different sequence identities. Sequence similarity as measured by sequence identity ranges from [0, 0.1) (least similar) to (0.9, 1.0] (most similar). **b**, TM-Vec absolute value of prediction error obtained from protein sequences compared with TM-scores from TM-align obtained from protein structures. Prediction errors were stratified across 681,000 proteins from three test benchmarking datasets: pairs, domains and folds. The pairs test dataset included protein sequence pairs that were left out of model training and/or validation. Similarly, the domains and folds test dataset included protein pairs derived from domains and folds that were never seen in model training and/or validation. Bounds of the boxplots denote 25% and 75% percentiles, the center is the 50% percentile and the whiskers denote the 1.5× interquartile range. **c**, t-SNE (*t*-distributed stochastic neighbor embedding) visualization of protein embeddings from the top five most represented categories from each CATH classification tier (class, topology, architecture, homology) within the test dataset. For each CATH classification tier, TM-Vec embeddings were observed to separate structural categories better than the default protein sequence embeddings generated by ProtTrans. **d**, Quantitative benchmarks of the ability of TM-Vec to predict CATH labels. We compared with ProtTrans and five structure-based methods: cliques, GRAFENE, ORCA, CNN (influenced by DeepFRI) and GCN (influenced by the Kipf and Welling GAE). Adjusted mutual information was computed by comparing spectral clustering assignments with structural label assignments for each CATH classification tier. Triplet-scoring AUPR is a metric that determines how often cosine embedding distances from within structural categories are smaller than cosine embedding distances across structural categories.

To evaluate the viability of our TM-score prediction strategy, we benchmarked TM-Vec on the SWISS-MODEL and CATH databases (Fig. 2), and compared our approach with multiple state-of-the-art structure-based and sequence-based methods. After training TM-Vec on approximately 150 million protein pairs from SWISS-MODEL (from 277,000 unique SWISS-MODEL chains), we observed a low prediction error (in the range of 0.025) that was independent of sequence identity across 1 million held-out protein pairs (Fig. 2a). Like traditional sequence alignment methodologies, TM-Vec can accurately estimate structural differences when the sequence identity is greater than 90% (median error = 0.005). Unlike traditional sequence alignment methods, which typically cannot resolve sequence differences below 25% sequence identity^46^, TM-Vec can resolve structural differences (and detect significant structural similarity) between sequence pairs with percentage sequence identity less than 0.1 (median error = 0.026). Overall, there was a strong correlation between the TM-scores predicted by TM-Vec and those produced by running TM-align (*r* = 0.97, *P* < 1 × 10^−5^) (Supplementary Fig. 4a).

We next validated TM-Vec on CATH protein domains that were clustered at 40% sequence similarity. For this, we validated predictions of TM-Vec on three CATH held-out datasets: (1) pairs (of domains) that were never seen in training together; (2) domains that were held out; and (3) folds that were held out. TM-Vec accurately predicted TM-scores for proteins from held-out pairs (*r* = 0.936, *P* < 1 × 10^−5^, median error = 0.023) as well as held-out domains (*r* = 0.901, *P* < 1 × 10^−5^, median error = 0.023) (Fig. 2b and Supplementary Fig. 4b). TM-Vec’s prediction errors were highest for pairs with TM-scores in the [0.75–1.0] range, and its accuracy declined on held-out folds. However, the incremental increase in the generalization error for proteins in the held-out folds (*r* = 0.781, *P* < 1 × 10^−5^, median error = 0.042) shows that TM-Vec is robust to out-of-distribution observations, a critical requirement for extrapolating beyond the experimental structures within the Protein Data Bank (PDB)^47^ (Fig. 2b and Supplementary Fig. 4b).

To further validate this finding, we applied the TM-Vec model trained on SWISS-MODEL chains to the Microbiome Immunity Project (MIP)^48^, which contains 200,000 de novo protein structure predictions from previously unknown proteins, including 148 putative folds. The correlation between our predictions and the TM-scores from MIP protein pairs with putative folds (*r* = 0.785, *P* < 1 × 10^−5^) was surprisingly close to the estimates we observed with the held-out CATH folds. Supplementary Table 1 shows a confusion matrix for our TM-score predictions for protein pairs where each protein has a putative fold; we observed that TM-Vec had a 99.9% true positive rate for predicting whether a pair shared a fold (TM-score ≥ 0.5) and a false positive rate of 3.9%. Taken together, these validation benchmarks across SWISS-MODEL, CATH and MIP show that TM-Vec is suitable for detecting similarities between proteins with previously unknown protein structures and folds, extending the general utility of this work.

### Capturing structural information in the latent space

We visualized and benchmarked the learned representations produced by TM-Vec against an array of alternative methods that depend on either sequence or structure alone. The results of our benchmarks show that TM-Vec implicitly learns representations that correlate well with structural classifications (Fig. 2). As shown in Fig. 2c, TM-Vec embeddings capture the latent structural features of the CATH hierarchy. For comparison, embeddings produced by ProtTrans^35^, the pretrained language model on which TM-Vec is based, are shown side by side with those of TM-Vec after training (Fig. 2c). The ProtTrans embeddings for proteins are calculated by averaging the ProtTrans per-residue embeddings. Across every tier of CATH, TM-Vec separates CATH structural classes more clearly than the default ProtTrans embeddings.

To further evaluate the structural information of TM-Vec protein vectors, we encoded the CATH database using TM-Vec and performed search and classification. In our search benchmarks, we observed that TM-Vec was able to correctly retrieve proteins with the same fold (topology level in CATH) in CATHS100 (97% accuracy) and CATHS40 (88.1% accuracy) for queried proteins (Supplementary Table 2). We next compared TM-Vec retrieval with FoldSeek (which performs its structure search on the ground truth CATH domain structures)^27^, MMseqs2 (which uses the CATH sequences)^49^, and another structure-aware protein embedding method, ProtTucker^29^, which is trained on CATH domain sequences and uses contrastive learning to learn domain representations (Supplementary Table 3). To make a head-to-head comparison with ProtTucker and these other methods, we trained a TM-Vec model on the same domains as ProtTucker’s model and evaluated TM-Vec on their test set of 219 domains (ProtTucker benchmark data). Across each level of the CATH hierarchy, TM-Vec outperformed FoldSeek, MMseqs2 and ProtTucker (Supplementary Table 3). At the homology level, TM-Vec retrieved proteins with 81% accuracy, ProtTucker (EAT) retrieved proteins with 78% accuracy and FoldSeek retrieved proteins with 77% accuracy. As this test set of 219 proteins was quite small, we chose to also compare these different methods on the CATHS20 dataset, alongside other methods including HHBlits^50^ and DIAMOND^51^ (Online methods). Here, the lookup and query databases were both CATHS20, and the TM-Vec model was the same model trained on the ProtTucker domains (Supplementary Table 4). Our evaluation criterion was the accuracy of retrieving the correct CATH homology for a query domain (which was itself excluded from the lookup database). Here, the TM-Vec model trained on CATH domains performed the best (88% accuracy), followed by FoldSeek (85%), ProtTucker (71%) and HHBlits (49%) (Supplementary Table 4). Notably, a TM-Vec model trained on SWISS-MODEL chains achieved 71% accuracy on this CATH domain benchmark.

In our classification benchmarks, we compared TM-Vec with several state-of-the-art methods (cliques, CNN, GCN, GRAFENE, ORCA and ProtTrans; Methods) using cluster-adjusted mutual information and triplet-scoring area under the precision–recall curve (AUPR) to assess the representation quality of each method (Methods). TM-Vec outperformed the sequence-based and structure-based methods for topology, homology and architecture classification as demonstrated by its higher macro AUPR values for these tiers, indicating that TM-Vec was convolving both sequence and structure knowledge bases (Fig. 2d). At the class level, cliques and CNN achieved higher macro AUPR values than TM-Vec. At the topology level, TM-Vec had the highest macro AUPR value (0.94), and the second best method was GRAFENE (macro AUPR = 0.79). The performance gap between the pretrained ProtTrans model (macro AUPR = 0.66) and the fine-tuned model obtained with TM-Vec highlights the importance of fine-tuning with a structure-based objective.

Furthermore, the fact that TM-Vec outperformed sequence-based representations on the CATH dataset that was clustered at 40% sequence similarity provides evidence that TM-Vec learns quality structural features rather than a trivial feature of the underlying data or a function of sequence similarity.

### Extracting structural alignments from sequence

We benchmarked DeepBLAST against three sequence alignment methods, Needleman–Wunsch^52^, BLAST^1^ and HMMER^2^, in addition to four structural alignment methods that work directly with the atomic coordinates, FAST^19^, TM-align^15^, Dali^18^ and Mammoth-local^20^ (Table 2). TM-align achieves global alignment by maximizing the three-dimensional (3D) spatial overlap of the atoms in each protein. Conversely, the Mammoth-local structure alignment scores feasible residue pairings between the proteins according to the structural similarity of seven-contiguous-neighbor windows, as opposed to a remote homology philosophy where the full length structure is allowed to be flexible and does not require all the aligned atoms to overlap simultaneously after a rigid body orientation. Dali uses a distance matrix computed from hexapeptide contacts to align the two protein structures. FAST tries to preserve similar residue–residue contact patterns. We extracted the local structure alignment from the first phase of the Mammoth algorithm. These structure alignment algorithms span the range of expert opinions as to the most meaningful structure alignment (from emphasizing long-range overlap to contacts and local similarity) and thus span potential disagreement across different previous approaches. No structure alignment algorithms tested took sequence similarity into account.

**Table 2:** Malisam and Malidup benchmarks

	Malidup		Malisam	
Method	F1 score	Number detected	F1 score	Number detected
BLAST	0.019 ± 0.019	5	0.000 ± 0.000	2
HMMER	0.020 ± 0.02	8	0.020 ± 0.020	3
Needleman–Wunsch	0.098 ± 0.010	234	0.025 ± 0.003	129
Smith–Waterman	0.114 ± 0.010	234	0.031 ± 0.003	129
DeepBLAST	**0.265** ± 0.020	234	**0.066** ± 0.009	129
Mammoth-local	0.483 ± 0.020	234	0.187 ± 0.017	129
FAST	0.569 ± 0.026	234	0.300 ± 0.030	129
TM-align	0.576 ± 0.024	234	0.393 ± 0.031	129
Dali	**0.791** ± 0.014	234	**0.619** ± 0.029	129

Sequence and structure alignment methods measured by their F1 score. FAST, TM-align, Dali and Mammoth-local are structure–structure alignment methods and provide a structure-informed upper bound for this benchmark, as many of the most challenging alignments in this benchmark are ultimately structure derived or curated with a structure–structure alignment as an oracle. The best F1 scores for sequence and structure alignment methods are highlighted in bold.

Our method DeepBLAST uses sequence alone; we do not supply the atomic coordinates of either protein to the algorithm after training it. To form a common reference for an optimal alignment, we focused on two gold-standard benchmark sets comprising manually curated structural alignments, named Malisam^44^ and Malidup^43^. Manual structure alignment is intuitive human assessment, typically emphasizing 3D overlap and topology preservation, as those features are easier to visualize than a plethora of local alignments and contacts^53–55^. All methods tend to agree when the problem is trivial owing to near sequence identity and near structural identity. Therefore, the most valuable gold-standard alignment benchmark set is where the dataset members have low sequence identity as well as varied degrees of structural similarity. Our benchmarks were performed on the curated Malisam^44^ and Malidup^43^ protein structural alignment benchmarking datasets (which are heavily skewed towards difficult-to-detect, low-sequence-identity remote homology).

As shown in Table 2, DeepBLAST outperformed all tested sequence alignment methods (Supplementary Fig. 3) but did not challenge the structural alignment methods. In both benchmarks, most of the protein alignments did not pass the filtering steps in both BLAST and HMMER. As a result, these tools were not able to detect the vast majority of the alignments. This left Needleman–Wunsch and Smith–Waterman as the baseline for sequence alignment methods. It is important to note again that there is no one definition of the best structural alignment^56,57^ and that this task becomes increasingly ambiguous as the remoteness of the homolog increases and the number of homologous residues declines. This was apparent in the Malidup benchmark, where the variation in differences between TM-Vec and TM-align as well as DeepBLAST and TM-align increased for proteins with TM-score < 0.5 (Supplementary Fig. 5a). Thus, the above F1 score tracks well with alignment accuracy but is limited in that it only scores sequence alignments with respect to a single reference alignment contained within the curated set.

### Remote homology detection and alignment

**Fig. 3:**
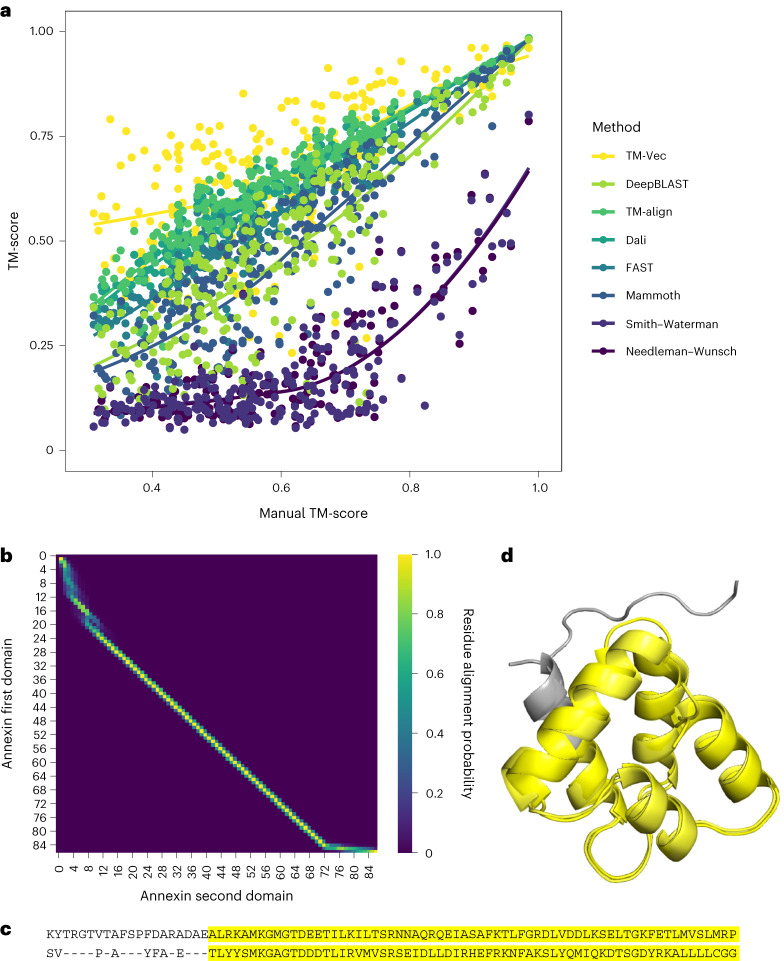
Annotating and aligning proteins in the Malidup benchmark. **a**, Comparison of different sequence and structural alignment methods with DeepBLAST and TM-Vec. DeepBLAST, Needleman–Wunsch and Smith–Waterman are sequence alignment methods, whereas Fast, Dali, Mammoth and TM-align are structural alignment methods. The *y* axis represents the predicted TM-score (for the alignment methods, this is given by a predicted alignment), and the *x* axis represents the TM-score from a manually curated alignment. The performance of TM-Vec was comparable with that of structural alignment methods, and its trend line overlapped with that of TM-align. The performance of DeepBLAST was similar to that of Mammoth, a structure alignment method, and it outperformed the other sequence alignment method, Needleman–Wunsch. Data are presented as mean values estimated with a locally estimated scatterplot smoothing fit with 95% confidence intervals. **b**, A predicted alignment of two duplicated Annexin domains from Malidup, where DeepBLAST could accurately align (TM-score = 0.81) and Needleman–Wunsch struggled to align (TM-score = 0.33). **c**, Manual alignment of the two duplicated Annexin domains; the agreement with DeepBLAST is highlighted. **d**, Visualization of the manual structural alignment of the Malidup; the chains that DeepBLAST aligned correctly are highlighted in yellow.

To gauge the performance of TM-Vec compared with existing structural alignment methods, we applied TM-Vec to the curated Malidup protein structural alignment benchmarking dataset^43^, a difficult benchmark with low sequence identity and varied degrees of structural similarity. Each pair of proteins in this benchmark has a significant structurally similar region, a manually curated structure–structure alignment, and low sequence similarity that is either below or at the threshold of detection by sequence alignment tools. One of the challenges of benchmarking structural alignment methods is defining the ground truth structural alignment. As shown in Fig. 3a, there were subtle disagreements between the manual alignments and the structural alignment methods, highlighting the uncertainty in defining the optimal structural alignment. This is highlighted in scenarios where TM-align obtains a better structural superposition compared with the manual alignment (TM-align superimposes more atoms, or a greater extent of backbone regions, than the manual alignment). All of the structure-aware methods agreed at high structural similarity, TM-score = 1 being perfect superposition of all atoms, but increasingly disagreed as the TM-score declined.

We observed that TM-Vec was directly comparable with structure-aware methods, and the confidence bands for its trend line overlapped with the trend line for TM-align (Fig. 3a). We also found that DeepBLAST was directly comparable with the structure-aware method Mammoth, as their trend lines and predictions were very similar. Although the trend lines overlapped, the prediction errors of TM-Vec and DeepBLAST had higher variance than those of the structure-aware methods. To determine the agreement between sequence alignment methods and structural alignment methods, the TM-score was calculated for the predicted alignment. Although DeepBLAST does not always generalize for divergent proteins, to illustrate an example where our method did obtain correct alignments for highly divergent proteins, we focused on two duplicated Annexin domains with a sequence identity of 24.7%. DeepBLAST accurately aligned these proteins (TM-score = 0.81) and four of the five folds that were superimposed were in agreement with the manual alignment (Fig. 3b–d). By contrast, Needleman–Wusnch was not able to identify any structural similarity between these two proteins (TM-score = 0.33). On the Malidup benchmark, the Spearman rank correlation between the DeepBLAST and TM-Vec TM-scores was 0.75, and the correlations for DeepBLAST and TM-Vec with TM-align’s TM-scores were 0.81 and 0.66, respectively (Supplementary Fig. 5).

The differences between Needleman–Wunsch and DeepBLAST were clear across all the protein pairs in Malidup and Malisam. Based on the percentage of structural similarity, given by the percentage of the smaller protein that aligns, scores shown in Supplementary Fig. 6, the high confidence alignments predicted by DeepBLAST were largely in agreement with the manually curated structural alignments. Furthermore, the sequence identity scores shown in Supplementary Fig. 6 indicate that DeepBLAST is able to obtain structural alignments for pairs that have ≤25% sequence identity, a known barrier for sequence alignment methods but one that can be resolved with the known protein structures. Taken together, these metrics suggest that DeepBLAST can perform local structural alignment.

### Full repository-level scaling and runtime

To show that TM-Vec can be applied to modern protein repositories, we benchmarked its search runtime in multiple scenarios. After the creation of a TM-Vec database, a query is performed for a new protein sequence by first encoding it using the TM-Vec model and then performing rapid vector search on the indexed protein TM-Vec database (Fig. 1). Search runtimes for different numbers of queries and database sizes (Supplementary Fig. 7) empirically show that encoding of queries is linear in time, with an ability to encode 50,000 queries on one GPU within 40 min (Supplementary Fig. 7a). Supplementary Fig. 7b shows sublinear search performance. The search runtime benchmarks for different database sizes show that 50,000 queries on a database of 5 million proteins can be performed within 20 s on a single GPU, demonstrating that encoding of sequences is the computational bottleneck in search. To contrast the TM-Vec query search time with that of existing sequence-based methods, we compared the TM-Vec query runtimes to those of DIAMOND^51^ and BLAST. TM-Vec was not as fast as DIAMOND, which is optimized for short-reads and is known to have remote homology sensitivity and alignment performance similar to BLAST. TM-Vec did outperform BLAST in all cases, including in modes adapted for scaling TM-Vec described here, and its performance will scale sublinearly with database size (Supplementary Fig. 7c). For example, TM-Vec achieved a 10× speedup compared with BLAST when performing 1,000 queries on a database of 100,000 proteins, and this speedup will increase exponentially as the database size increases: on a 1 million protein database there is a 100× speedup.

The development of TM-Vec overcomes two major challenges to applying structural alignments predicted from DeepBLAST at scale: avoiding all-versus-all pairwise comparisons and predicting structural similarity. Thus, TM-Vec can be used to carry out full repository searches and large all-versus-all queries, and can do so with vastly improved remote homology detection and sensitivity. Further gains in computational performance are likely to be achievable (this work focuses on accuracy and sensitivity with respect to structure–structure quality alignments).

Once structurally similar proteins have been identified, structural alignments via DeepBLAST can identify structurally homologous regions. Our structural alignment runtime benchmarks show that unlike the Needleman–Wunsch CPU implementations, the structural alignment runtime of our differentiable Needleman–Wunsch GPU implementation does not increase linearly with respect to the batch size, demonstrating how our method can process multiple alignments in parallel on a single GPU (Supplementary Fig. 7d). Furthermore, both the CPU and GPU implementations scale linearly with regard to the length of both proteins, with our GPU implementation consistently yielding a 10× speedup over the CPU implementation.

As shown in Supplementary Table 5, we further evaluated the ability of TM-Vec to scale to full repositories and achieve competitive results by evaluating its performance on the DIAMOND benchmark (Methods), which has UniRef50 (ref. ^16^) as a lookup database and comprises both single-domain and multiple-domain proteins. For this benchmark, we used the TM-Vec model trained on SWISS-MODEL chains up to 1,000 residues long. DIAMOND has a sensitivity of 99% for the top protein on this benchmark (for all proteins). After embedding proteins in the UniRef50 lookup database, we compared our performance on all query proteins versus only multiple-domain proteins, and on proteins with different length thresholds (600 and 1,000 residues). For all proteins up to 1,000 residues long, the top nearest neighbor shared the same family annotation 92.1% of the time, and among the top 50 nearest neighbors, the sensitivity was 96.9% (Supplementary Table 5). For only multiple-domain proteins, the top nearest neighbor shared the same family annotations 86.2% of the time for proteins up to 600 residues long, and 82.6% of the time for proteins up to 1,000 residues long. Among the top 50 returned proteins, the sensitivity was 94.6% for multiple-domain proteins up to 1,000 residues long (Supplementary Table 5). When returning many nearest neighbors (Supplementary Table 6) on one GPU, once vectors were available, TM-Vec could efficiently return 1 million nearest neighbors for 100 queries on a lookup database of 10 million proteins in about 1 s. In terms of resources, we could index and search over UniRef50 (ref. ^16^) on one GPU, but as repositories scale to billions of proteins, multiple-GPU or high-memory CPU setups using Faiss^41^ are recommended for running a TM-Vec + DeepBLAST pipeline.

### Case study: bacteriocins

We analyzed a structurally diverse set of families, bacteriocins, using the BAGEL database^45^. Bacteriocins are small peptide-derived molecules produced by bacteria and often serve as antimicrobial peptides to target competing microbial species. They can also be involved in cell–cell communication. Several bacterial species encode bacteriocins, and bacteria are under evolutionary pressure to obfuscate these genes in light of their strong ecological benefits. As a result, bacteriocins show substantial sequence and structural diversity and are notoriously difficult to detect using sequence homology tools^58^. To date, fewer than 1,004 bacteriocins have been identified and classified, despite there being trillions of microbial species^59^ that have the potential to produce antimicrobial peptides.

**Fig. 4:**
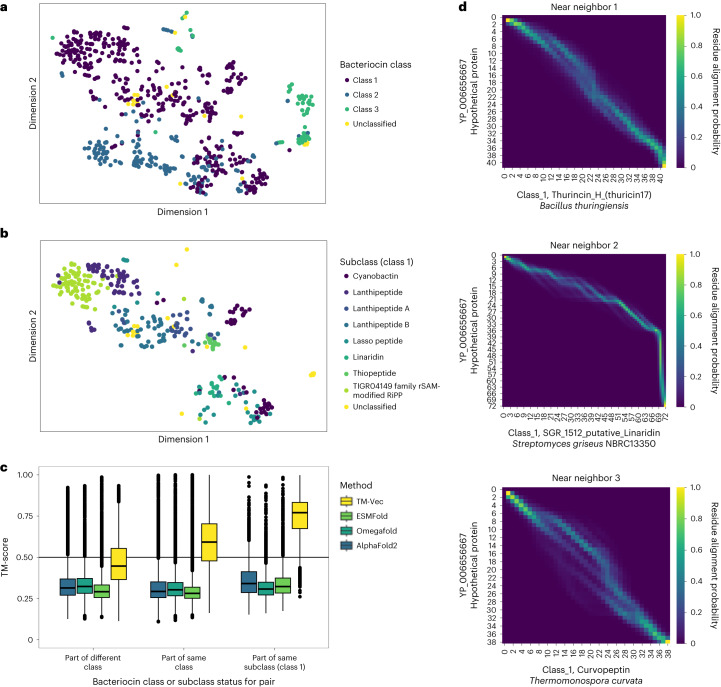
Annotating and aligning unclassified putative bacteriocins using TM-Vec. **a**, Visualization of TM-Vec embeddings using t-SNE for 689 proteins across three classes of bacteriocins in addition to 28 unclassified putative bacteriocins. For 94% of the annotated bacteriocins, the nearest neighbor to a classified bacteriocin was in the same bacteriocin class. **b**, Visualization of class 1 bacteriocins by subclass, highlighting how TM-Vec can recover multiple levels of manual annotation without protein structures. **c**, Comparison of TM-Vec’s TM-score predictions with the TM-scores produced by running TM-align on structures predicted by AlphaFold2, OmegaFold and ESMFold for 238,000 pairs of bacteriocins. Using a TM-score of 0.5 as a structural similarity cutoff, TM-Vec could distinguish pairs of proteins that were in the same class versus different classes and in the same subclass for class 1 bacteriocins, whereas TM-align on predicted structures from AlphaFold2, OmegaFold and ESMFold could not. Bounds of the boxplots denote 25% and 75% percentiles, the center is the 50% percentile and the whiskers are denoted by the 1.5× interquartile range. **d**, DeepBLAST alignments for a putative bacteriocin, YP_006656667, and its three nearest neighbors in embedding space (that is, those with the highest predicted TM-scores).

Previous studies have shown that bacteriocin structures can be characterized by their highly modified polypeptides, suggesting structural cues to identify new bacteriocins where sequence similarity approaches fail. Our analysis revealed that TM-Vec can clearly partition bacteriocins according to their BAGEL annotations (Fig. 4a,b). Notably, unannotated bacteriocins identified by Morton et al.^58^ were found to be structurally similar to lanthipeptide A and B (Fig. 4b). As shown in Fig. 4c, we compared TM-Vec with AlphaFold2 (ref. ^10^), OmegaFold^39^ and ESMFold^40^ on this bacteriocin dataset. For each pair of bacteriocins, we ran TM-align on the structures predicted by each structure prediction method (AlphaFold2 via ColabFold^11^, OmegaFold and ESMFold). We found that TM-Vec distinguished bacteriocin classes more accurately than these structure prediction methods in combination with TM-align. We suspect that this performance gap could be due to the lengths of the proteins. Bacteriocins tend to be short sequences of fewer than 50 amino acids, which are known to be difficult to fold using AlphaFold2 (ref. ^26^). For a few of the bacteriocins with structures in the PDB, we found that the structures predicted by AlphaFold2 had TM-scores < 0.5 with the ground truth structures, highlighting how AlphaFold2 struggles to accurately predict bacteriocin structures. The performance of the structure prediction methods on this benchmark is likely to result from a combination of inaccurate bacteriocin structure predictions and the effects of applying structure alignments (TM-align) to predictions (structure predictions)^60^. Last, Fig. 4d shows a DeepBLAST alignment for the three nearest classified bacteriocin neighbors of a putative bacteriocin identified by Hamid et al.^61^.

As shown in Supplementary Fig. 8a, we found that nontoxins (that is, genes that are within the same biosynthetic gene cluster but do not directly encode the toxin) were clearly separated from the different bacteriocin class clusters based on structural similarity. We further tested our ability to distinguish bacteriocins by training a *k*-nearest-neighbor classifier for bacteriocin classes and nontoxins; the overall precision and recall of these classifiers were 98% and 93%, respectively (Supplementary Fig. 8b).

## Discussion

We have shown that DeepBLAST and TM-Vec have the potential to close the remaining gap between protein sequence and structural information by enabling structural alignments from sequence information and remote homology search on repository-scale protein sequence databases. On our SWISS-MODEL and CATH benchmarks, TM-Vec can accurately predict TM-scores to quantify the structural similarities across widespread structural diversity, including remote homologs that fall below the 10% sequence identity threshold. When compared with sequence-based and structure-based methods, TM-Vec could competitively differentiate tiers of the CATH hierarchy, despite not being explicitly trained to classify CATH classes. Furthermore, TM-Vec is able to predict structural similarity with performance close to that of existing structural similarity methods, while being able to query structurally similar proteins with both higher accuracy and lower runtimes than BLAST. TM-Vec search scales sublinearly with respect to protein database size and can handle millions of queries on tree-of-life-scale databases per day on a single GPU machine. Given the runtime scaling properties of TM-Vec, there is enormous potential to apply these methodologies to large-scale metagenomics datasets. However, realizing the full potential of TM-Vec will require improvements to encoding speed in addition to massively parallel GPU computing in order to query hundreds of millions of proteins in metagenomics samples and billions of proteins in sequence databases.

In addition to TM-Vec measuring structural similarity, DeepBLAST can provide structural alignments that compare with existing structural alignment methods. On the Malidup benchmark, although there were certain remote homologs that our aligner missed, DeepBLAST consistently outperformed sequence alignment methods. When we applied TM-Vec to the BAGEL database, we were able to accurately cluster bacteriocins based on both their class and subclass labels, a task that AlphaFold2 struggles with. We also were able to confidently annotate 28 putative bacteriocins by finding their nearest class or subclass clusters. These results hint at the potential to lower the barrier for natural product discovery.

Although TM-Vec and DeepBLAST have promising advantages compared with existing methodologies, there are a few limitations to consider. TM-Vec is not well suited to detection of structural differences induced by point mutations. From a benchmark using the VIPUR dataset, TM-Vec was unable to detect structural differences caused by both deleterious and synonymous point mutations in proteins^14,62–66^. TM-Vec is trained to predict TM-scores, which are a measure of the global similarity of protein structures. For many remote homology tasks, local similarity is instead desired. On the DIAMOND benchmark, for example, the goal of retrieving proteins with the same SCOP family annotations is more of a local than a global similarity task. On this benchmark, although TM-Vec had high sensitivity, it did not perform as well as DIAMOND, suggesting that there is room to improve TM-Vec on this task if TM-Vec is trained with a local structural similarity objective instead of TM-scores^67^. Regarding structural alignments, DeepBLAST struggles to detect large insertions or deletions, which are commonly observed in remote homologs as suggested by TM-align generated training data. Recent advances incorporating linear affine gap costs into differentiable dynamic programming algorithms^28^ could play a part in resolving these challenges. Furthermore, integrating the TM-score prediction and the structural alignments into a multitask framework with a single pretrained protein language model may help to boost the structural alignment accuracy.

Given the widespread biomedical applications and use cases of sequence search and alignment using tools such as BLAST, we anticipate that structural similarity search with TM-Vec and alignment with DeepBLAST will provide new opportunities for biological annotation. Owing to their high structural precision and fast query speed, TM-Vec and its future iterations are well positioned to close the sequence–structure–function gap across the billions of observed proteins.

## Methods

We used our model, which can produce structure-aware embeddings of protein sequences, to build large, searchable databases of protein representations that can be queried to find proteins with similar structures using only their sequence information. The last piece of our pipeline produces protein structure alignments using sequences alone for the proteins that are predicted to have the most similar structures.

### TM-Vec search

#### TM-Vec embedding model

The TM-Vec model is trained on pairs of protein sequences and their TM-scores (the measure of protein structure similarity we use), and leverages the latest advances in deep protein language models. When protein sequences are fed into the pipeline, a pretrained deep protein language model ProtTrans (ProtT5-XL-UniRef50) is used to produce embeddings for every residue in the protein^35^. These residue embeddings are then fed into a twin neural network that we train, called *ϕ*. Supplementary Fig. 1 shows the function *ϕ* which takes residue embeddings and produces a flattened vector representation of dimension 512 for each protein. *ϕ* is composed of several transformer encoder layers (see the TM-Vec training section for transformer details), followed by average pooling, dropout and fully connected layers. Finally, we calculate the cosine distance between the reduced representations of each protein in the pair, and our training objective is to minimize the L1 distance between the cosine similarity of the reduced representations of the pairs and their TM-score. Therefore, for any pair of protein sequences, a forward pass of our model can predict the TM-score of the pairs, and can also be used to produce structure-aware embeddings for each protein sequence.

#### TM-Vec database creation

To build a large database of structure-aware protein embeddings, we started with large databases of protein sequences, including SWISS-Prot^68^, CATH^37^ and UniRef50 (ref. ^16^). After encoding each protein sequence, we built an indexed vector-searchable database of protein embeddings using the Faiss package^41^. When this database was queried with a new sequence, we first embedded the protein using a forward pass of the TM-Vec embedding model and then returned the nearest neighbors of the query according to cosine similarity (the proteins in our database with the highest predicted structural similarity or TM-score). Although one of our goals was to return the nearest neighbors in structure space for any query proteins, another goal was to include the structural alignments for the nearest neighbors with the query protein, using sequences alone. Thus, the predicted most similar proteins (structurally), their predicted TM-scores and their predicted structural alignments can all be returned by the TM-Vec + DeepBLAST pipeline, and the number of proteins for which to retrieve this information is a user-defined parameter (the pipeline will return the user-defined top *n*).

#### DeepBLAST alignment module

The DeepBLAST module uses a differentiable Needleman–Wunsch algorithm (Supplementary Fig. 2). Proteins *X* and *Y* are fed into the pretrained protein language model ProtTrans^35^ to obtain embeddings *H*_*X*_ and *H*_*Y*_. These residue-level embeddings are then propagated through the match embeddings (*M*) and gap embeddings (*G*) to obtain the match scores *μ* and the gap scores *g*. The match and gap scores are used to evaluate the differentiable dynamic programming algorithm and generate a predicted alignment traceback. These alignments can then be fine-tuned using a training dataset of ground truth alignments.

##### Protein language modeling for alignment

To obtain an alignment from dynamic programming, scoring parameters for matches and gaps must be obtained. Here, we use a number of pretrained protein language models to estimate these scoring parameters. These pretrained models ultimately construct a function, mapping a sequence of residues represented as one-hot encodings to a set of residue vectors, providing an alternative representation of these proteins. Often, these models will learn the representations by being trained to predict randomly masked residues within a protein sequence. Multiple studies have shown the merits of these models when performing protein structure prediction, remote homology and protein design^31–36,69^. Here, we use the pretrained ProtTrans language model^35^ to represent two proteins *X* and *Y* by embeddings ${{{H}_{X}}}\in {{\mathbb{R}}}^{p\times d}$ and ${{{H}_{Y}}}\in {{\mathbb{R}}}^{q\times d}$, where *p* and *q* represent the lengths of proteins *X* and *Y*, and *d* is the embedding dimension of the language model. Given these representations, we can construct mappings *M* and *G* to obtain match scores and gap scores for the differentiable dynamic programming as follows

$${{\mu }}={\sigma }_{\mu }\left(M({{{H}_{X}}}) M{({{{H}_{Y}}})}^{T}\right)\in {{\mathbb{R}}}^{p\times q}, \quad {{g}}={\sigma }_{g}\left(G({{{H}_{X}}}) G{({{{H}_{Y}}})}^{T}\right)\in {{\mathbb{R}}}^{p\times q}$$

The functions $M:{{\mathbb{R}}}^{t\times d}\to {{\mathbb{R}}}^{t\times d}$ and $G:{{\mathbb{R}}}^{t\times d}\to {{\mathbb{R}}}^{t\times d}$ are intermediate functions that take as input a set of *t* residue vectors. These functions are parameterized by convolutional neural networks, which can be fine-tuned through the backpropagation enabled by the differentiable dynamic programming. Activation functions *σ*_*μ*_ and *σ*_*g*_ are softplus and log-sigmoid functions to ensure that the match scores *μ* are strictly positive and the gap scores *g* are strictly negative. These constraints are used to penalize gaps and reward matches. This also helps enforce identifiability of the model, which we have found to improve the accuracy of the model in practice.

##### Differentiable dynamic programming

Our proposed differential dynamic programming framework does not learn any parameters; it is designed purely to enable backpropagation to fine-tune the scoring functions *M* and *G*. Differentiable dynamic programming has been extensively explored in the context of dynamic time warping^70,71^. Koide et al.^72^ and Ofitserov et al.^73^ suggested that a differentiable Needleman–Wunsch alignment algorithm could be derived, but the implementation has remained elusive. Here, we provide a GPU-accelerated implementation of the differentiable Needleman–Wunsch algorithm.

Previous work^71^ has shown that backpropagation can be performed on dynamic programming algorithms by introducing smoothed maximum and argmax functions. Doing so will enable the computation of derivatives while providing a tight approximation to the optimal dynamic programming solution. The traditional Needleman–Wunsch algorithm can be defined with the following recursion

1 $${v}_{i,\,j}={\mu }_{i,\,j}+{\mathrm{max}}\left\{\begin{array}{ll}{v}_{i-1,\,j-1}&{\mathrm{(Match)}}\\ {g}_{i,\,j}+{v}_{i-1,\,j}&{\mathrm{(Insert)}}\\ {g}_{i,\,j}+{v}_{i,\,j-1}&{\mathrm{(Delete)}}\end{array}\right.$$

where the alignment score *v*_*i*, *j*_ is evaluated on position *i* in the first sequence *X* and on position *j* in the second sequence *Y*. Sequences *X* and *Y* are of lengths *n* and *m*, respectively. *μ*_*i*, *j*_ represents the log-odds score of residues *X*_*i*_ and *Y*_*j*_ being aligned and *g*_*ij*_ represents the log-odds score of an insertion or a deletion at positions *i* and *j*. Owing to the structure of dynamic programming problems, *v*_*n*,*m*_ is guaranteed to be the optimal alignment score between the two sequences. Furthermore, the optimal alignment can be obtained by tracing the highest-scoring path through the alignment matrix via argmax operations.

As neither the max nor the argmax operations are differentiable, the alignment scores and the traceback cannot be differentiated in the traditional formulation of the traceback operations needed to generate alignments. Accordingly, Mensch et al.^71^ introduced smoothed differentiable operators

$${\tilde{max}}=\log \left(\mathop{\sum}\limits_{i}\exp ({x}_{i})\right),\,{\mathrm{argma{x}}}_{{{\Omega }}}(x)=\frac{\exp ({{{\boldsymbol{x}}}})}{{\sum }_{i}\exp ({x}_{i})}$$

where the smooth max operator $\tilde{max}$ is given by the log sum exp function and the smoothed argmax_Ω_(*x*) is given by the softmax function. As the softmax function can be derived from the derivative of max_Ω_, the traceback matrix can also obtained by differentiating the resulting alignment matrix. The resulting traceback matrix will yield the expected alignment between the two proteins.

As the loss function is defined as the difference between the predicted traceback matrix and the ground truth traceback matrix, the derivatives of the traceback matrix also need to be defined. This requires both the computations of the directional derivatives and the local Hessians of the alignment matrix (Algorithm 2).

In practice, dynamic programming can be a major computational bottleneck owing to GPU data transfer and the quadratic runtime of the Needleman–Wunsch algorithm. To address this, we implemented a GPU-accelerated differentiable Needleman–Wunsch algorithm inspired by Manavski et al.^74^. As can be seen from the benchmarks shown in Supplementary Fig. 7d, this algorithm is an order of magnitude faster than the naive CPU-bound Needleman–Wunsch implementation. Furthermore, this algorithm enables batching, allowing multiple alignments to be processed in parallel. As shown in Supplementary Fig. 7d, larger batch sizes can further improve the scaling compared with CPU-bound alignments.

**Algorithm 1.** Compute DeepBLAST_Ω_(*θ*)and ∇ DeepBLAST_Ω_(*θ*)

Require:$$\theta =[\mu ,g]\in {{\mathbb{R}}}^{2\times p\times q}$$

 Forward pass

 $${v}_{0,0}^{M}=1;{v}_{0,.}^{* }=0;{v}_{.,0}^{* }=0$$

 for *i* ∈ {1…*p*}, *j* ∈ {1…*q*} do

  $${v}_{i,\,j}={\mathrm{ma{x}}}_{{{\Omega }}}\ \left({\mu }_{i,\,j}+({v}_{i-1,\,j-1},{g}_{i,\,j}+{v}_{i-1,\,j},{g}_{i,\,j}+{v}_{i,\,j-1})\right)$$

  $${\omega }_{i,\,j}=\nabla {\mathrm{argma{x}}}_{{{\Omega }}}\ \left({\mu }_{i,\,j}+({v}_{i-1,\,j-1},\ {g}_{i,\,j}+{v}_{i-1,\,j},\ {g}_{i,\,j}+{v}_{i,\,j-1})\right)\in {{\mathbb{R}}}^{3}$$

 end for

 Backward pass

 *e*_*p*,*q*+1_ = 0; *e*_*p*+1,*q*_ = 0; *e*_*p*+1,*q*+1_ = 1

 for *i* ∈ {*p*…1}, *j* ∈ {*q*…1} do

  $${e}_{i,\,j}={\omega }_{i+1,\,j+1}^{m}{e}_{i+1,\,j+1}+{\omega }_{i+1,\,j}^{x}{e}_{i+1,\,j}+{\omega }_{i,\,j+1}^{y}{e}_{i,\,j+1}$$

 end for

 $W={(\omega )}_{i,\,j,k = 1}^{p+1,q+1};\ E={(e)}_{i,\,j = 1}^{p+1,q+1}$ 7D2; intermediate computations to be used in Algorithm 2

 return $${{{{\rm{DeepBLAST}}}}}_{{{\Omega }}}(\theta )={v}_{p,q},\nabla {{{{\rm{DeepBLAST}}}}}_{{{\Omega }}}(\theta )={(e)}_{i,\,j = 1}^{p,q}$$

**Algorithm 2.** Compute 〈 ∇ DeepBLAST_Ω_(*θ*), *Z*〉 and ∇^2^DeepBLAST_Ω_(*θ*)*Z*

Require $$\theta =[\mu ,g]\in {{\mathbb{R}}}^{2\times p\times q},Z=[{z}_{\mu },{z}_{g}]\in {{\mathbb{R}}}^{2\times p\times q}$$

 Forward pass

 *v*_0,0_ = 1; *v*_0,._ = 0; *v*_.,0_ = 0

 for *i* ∈ {1…*p*}, *j* ∈ {1…*q*} do

  $${\dot{v}}_{i,\,j}={z}_{{\mu }_{i,\,j}}+{\omega }_{i,\,j}^{m}({v}_{i-1,\,j-1})+{\omega }_{i,\,j}^{x}({z}_{{g}_{i,\,j}}+{v}_{i-1,\,j})+{\omega }_{i,\,j}^{y}({z}_{{g}_{i,\,j}}+{v}_{i,\,j-1})$$

  $${\dot{\omega }}_{i,\,j}\!=\!-{J}_{{{\Omega }}}({\omega }_{i,\,j})\left({\omega }_{i,\,j}^{m}({\dot{v}}_{i-1,\,j-1}),\ {\omega }_{i,\,j}^{x}({z}_{{g}_{i,\,j}}\!+\!{\dot{v}}_{i-1,\,j}),\!\ {\omega }_{i,\,j}^{y}({z}_{{g}_{i,\,j}}\!+\!{\dot{v}}_{i,\,j-1})\right)\in {{\mathbb{R}}}^{3}$$

 end for

 Backward pass

 *e*_*p*,*q*+1_ = 0; *e*_*p*+1,*q*_ = 0; *e*_*p*+1,*q*+1_ = 1

 for *i* ∈ {*p*…1}, *j* ∈ {*q*…1} do

  $${\dot{e}}_{i,\,j}={\dot{\omega }}_{i+1,\,j+1}^{m}{e}_{i+1,\,j+1}+{\omega }_{i+1,\,j+1}^{m}{\dot{e}}_{i+1,\,j+1}$$

   + $${\dot{\omega }}_{i+1,\,j}^{x}{e}_{i+1,\,j}+{\omega }_{i+1,\,j}^{x}{\dot{e}}_{i+1,\,j}$$

   + $${\dot{\omega }}_{i,\,j+1}^{\,y}{e}_{i,\,j+1}+{\omega }_{i,\,j+1}^{y}{\dot{e}}_{i,\,j+1}$$

 end for

 return $$\langle \nabla {{{{\rm{DeepBLAST}}}}}_{{{\Omega }}}(\theta ),Z\rangle ={\dot{v}}_{p,q},{\nabla }^{2}{{{{\rm{DeepBLAST}}}}}_{{{\Omega }}}(\theta )Z={(\dot{e})}_{i,\,j = 1}^{p,q}$$

##### Alignment loss function

By defining a loss function between the predicted alignment and the structural alignment from TM-align, we can evaluate the accuracy of DeepBLAST and fine-tune the functions *M* and *G*. Mensch et al.^71^ proposed using the Euclidean distance between the predicted and ground truth alignments as a loss function. However, we found that a cross-entropy loss provided more reasonable alignment results. This loss is given by

2 $$L({e}^{* },e)=\mathop{\sum}\limits_{i,\,j}{e}_{i,\,j}^{* }\log ({e}_{i,\,j})+(1-{e}_{i,\,j}^{* })\log (1-{e}_{i,\,j})$$

where e* is the ground truth alignment and *e* is the predicted alignment. As shown by Mensch et al.^71^, the predicted traceback matrix represents the expectation across all possible predicted alignments, which is represented as a matrix of probabilities. As a result, the resulting alignment problem can be interpreted as a classification task to identify whether two residues between a pair of proteins are alignable. This provides additional motivation for using cross-entropy as a loss function.

### Datasets

#### TM-Vec search

TM-Vec was trained on pairs of protein–domain sequences, along with data about the structural alignment for the pair. For every pair of proteins in our training dataset, we ran the method TM-align, which is an algorithm for protein structure comparison that is independent of protein sequences. TM-align produces a TM-score between 0 and 1, where a score below 0.2 represents a pair of unrelated proteins; a score above 0.5 implies that proteins are in the same fold; and 1 is a perfect match, indicating the same protein structure. Part of our pipeline involved validating whether our model could predict the TM-scores of pairs of proteins.

#### Protein-chain-pairs dataset

The model that we ultimately used to encode protein sequences was trained on pairs of protein chains. We sampled pairs of chains from SWISS-MODEL, which contains more than 500,000 chains. We made two different protein-chain-pair datasets, one with protein chains up to 300 residues long, and another with protein chains up to 1,000 residues long. For example, when we filtered out protein chains that were longer than 300 residues, we were left with 277,000 chains. With these chains in hand, we made pairs of chains, ensuring that we oversampled pairs of proteins with similar folds, using information from Gene3D^75^ about the predicted domains within protein chains. For all our pairs of protein chains, we ran TM-align using their SWISS-MODEL structures. We pulled out the TM-scores and sequence identity for every pair of chains. Last, we split our dataset into training, validation and test sets. For the chain-pairs dataset with chains up to 300 residues long, our train/validation split (randomly split during training) had 141 million pairs, and our held-out test dataset had 1 million pairs. Our chain-pairs dataset with chains up to 1,000 residues long had 320 million pairs.

#### Domain-pairs dataset

To determine whether our model could approximate TM-scores for domains and remote homologs, we built a dataset of pairs from the heavily curated CATH domains dataset. We started with the CATH nonredundant dataset of protein domains with no more than 40% sequence similarity. This dataset comprised 31,000 protein domains. We then filtered out domains that were longer than 300 residues, leaving 30,000 domains. All pairwise combinations of these 30,000 domains would lead to 450 million pairs; however, we aimed to build a balanced dataset, and dissimilar protein structures represented the vast majority of pairs (that is, domains with very different folds). Therefore, we undersampled pairs of CATH domains that came from different folds. The CATH dataset that we used for our experiments included 23 million pairs of domains.

We further split this dataset into training/validation and testing splits, and we evaluated performance on CATHS40 on left-out domain pairs (where the domain pair was not in the training/validation dataset), left-out domains (either one or both domains not in the training/validation dataset) and left-out folds (either one or both domains from folds that were not in the training/validation dataset). Here, the fold family was from the topology classification in the CATH hierarchy. Our training/validation dataset contained 19 million pairs, our left-out pairs dataset contained 100,000 pairs, our left-out domains dataset contained 100,000 pairs, and our left-out folds dataset contained 500,000 pairs.

#### Malidup and Malisam datasets

Some of our sequence alignment benchmarks were performed on the curated Malisam^44^ and Malidup^43^ protein structural alignment benchmarking datasets. All the structural alignments analyzed were provided from the original benchmark^43,44^. We also used Malidup to benchmark TM-Vec and DeepBLAST. Malidup consists of 241 pairwise structure alignments for homologous domains within the same chain. These pairs are structurally similar remote homologs. Malisam consists of 130 pairs of analogous motifs.

#### Structure alignment dataset

We trained DeepBLAST on 1.5 million alignments from the PDB^47^ obtained using TM-align^15^. These proteins were obtained from a curated collection of 40,000 protein structures^76^. Details of the model specification and training can be found in ref. ^77^.

#### Bacteriocins dataset

The bacteriocin sequences and metadata we used were from the bacteriocin database BAGEL^45^, and the putative unannotated bacteriocins were from Morton et al.^58^.

#### MIP novel fold dataset

In this project, there were protein structure predictions for 200,000 diverse microbial protein sequences, representing 148 putative novel folds, and the authors calculated TM-scores for pairs of proteins with novel folds^48^. We evaluated our TM-score predictions on 184,000 pairs of MIP proteins for which at least one protein in the pair had a novel fold.

#### ProtTucker benchmark dataset

ProtTucker was built to embed protein domains in a structure-aware way and uses CATH domains for its contrastive learning approach^29^. For this benchmark, we followed the ProtTucker training–lookup–test splits for the purpose of direct comparison with their method. Their training and lookup datasets consisted of 66,000 and 69,000 CATH domains, respectively. The test dataset did not include any domains with an HSSP-value > 0 with any of the lookup domains^78^ and consisted of 219 domains. We created a domain-pairs dataset from their set of 66,000 training domains in the same manner as our other CATH domain-pairs dataset by sampling pairs of domains and then running TM-align to produce TM-scores for the pairs. Our final training dataset included 35 million domain pairs.

#### DIAMOND benchmark dataset

The DIAMOND benchmark^51^ consisted of a large query dataset and a large lookup dataset of single and multidomain proteins. The lookup dataset was from the 14 September 2019 release of UniRef50 (ref. ^16^), which contained 37.5 million sequences; the authors then reduced this to a representative dataset of 7.74 million protein sequences with protein family annotations (SCOP)^53^. The query dataset was from the 25 October 2019 release of the NCBI nr database and also used the SCOP family annotations for proteins; the authors reduced this dataset to include at most 1,000 protein sequences for each SCOP superfamily, resulting in a dataset of 1.71 million queries. Finally, the authors locally shuffled both the query and the lookup sequences in this benchmark in 40-letter windows outside their annotation ranges.

#### Embedding methods data

For this evaluation we used the CATH NR-S40 dataset (NR-S40) (ref. ^37^), a collection of approximately 30,000 proteins of maximally 40% sequence identity, representing a diverse sampling of each tier in the CATH hierarchy. The dataset was partitioned into training, validation and test sets. All the benchmarks were conducted on the test set, and all trainable methods in the comparison study were trained using the training and validation sets.

### TM-Vec training

The TM-Vec models trained on CATHS40 and SWISS-MODEL chains up to 300 residues long both had 17.3 million trainable parameters and were 199MB in size. These models contained two transformer encoder layers. The TM-Vec models trained on CATHS100 domains (ProtTucker training domains) and SWISS-MODEL chains (up to 1,000 residues long) both had 34.1 million trainable parameters and were 391 MB in size. These models contained four transformer encoder layers.

The pretrained deep protein language model that we used, ProtTrans (ProtT5-XL-UniRef50), had no trainable parameters in our pipeline (the model parameters were frozen), as we used the model exclusively for extracting residue embeddings with a dimension of 1,024. Our transformer encoder layers had four multihead attention heads and a dimension of 2,048 in their feedforward network model. We used the Adam optimizer to train the weights, with an initial learning rate of 1 × 10^−4^. A batch size of 32 was used. In terms of training requirements, for the TM-Vec model trained on SWISS-MODEL chains up to 300 residues long, we trained TM-Vec on eight Nvidia V100 GPUs for 5 days. This represented five epochs of training.

### DeepBLAST training

The final DeepBLAST model consisted of eight convolutional layers of dimension 1,024 to parameterize the match embeddings *M* and gap embeddings *G*. We used the same ProTrans model to estimate residue vectors. The resulting model had more than 1.2 billion parameters. We used the Adam optimizer to train the weights, with an initial learning rate of 5 × 10^−4^, and the pretrained model weights were frozen. A batch size of 360 alignments was used for training. DeepBLAST was trained for 20 epochs on 24 Nvidia A100 GPUs for 6 days. The DeepBLAST model was trained on a dataset of 5 million alignments obtained from TM-align. Alignments containing more than 10 consecutive gaps or with TM-score less than 0.6 were excluded from the training dataset.

### DeepBLAST alignment accuracy assessment

Alignment accuracy was assessed on a held-out test dataset of 1 million structural alignments. Validation loss was recorded during training, and we stopped training once the validation loss stopped decreasing (Supplementary Fig. 9). To determine how well DeepBLAST generalizes, a subset comprising more than 120,000 alignments that were in the held-out TM-align alignments used to train DeepBLAST were analyzed. To evaluate the accuracy of the alignments, precision and recall were computed from the number of correctly identified matching residues. As each alignment can be represented as a bipartite graph where the edges represents matching residues between two proteins, precision and recall can be extracted by comparing the edge sets of the predicted alignment and the known alignments. Supplementary Fig. 9 shows the distribution of correctly identified alignment edges, with a median recall and precision of 87%, suggesting that these models can generalize well beyond the training dataset.

### DIAMOND benchmark

The metric that we used to evaluate the performance of our method on the DIAMOND benchmark was sensitivity, which we defined as the percentage of the time the family annotations of the query protein were among the family annotations of the returned top *n* nearest neighbor proteins. For example, for the top 10 nearest neighbors, this quantifies the percentage of the time that the family annotations of the query protein are included in the family annotations of the returned top 10 nearest neighbor proteins.

### Bacteriocin benchmark

We compared TM-Vec with three structure prediction methods for this benchmark: AlphaFold2, ESMFold and OmegaFold. ColabFold^11^ was used to run AlphaFold2 (ref. ^10^) using default parameters and the MMseqs2 pipeline. ESMFold v.1 was used for ESMFold structure predictions, and OmegaFold model 1 was used for OmegaFold structure predictions.

### Embedding methods benchmarks

As shown in Fig. 2b, we compared TM-Vec with six other representations: one sequence-based method, ProtTrans^35^; and five different structure-based methods: cliques, GRAFENE^79^, ORCA^80^, CNN (influenced by DeepFRI^81^) and GCN (influenced by the Kipf and Welling graph autoencoder (GAE))^82^. Each structure-based method in some manner consumes a thresholded distance matrix, or contact map, and is used to output a fixed-sized feature vector that is meant to encode structural information.

The structure-based methods cliques, GRAFENE and ORCA output so-called manually engineered features; in particular, these feature vectors are histograms over known nonredundant graph substructures called graphlets. We introduce cliques as a simple baseline that consists of counting the ratio of nonoverlapping cliques up to size 7 inside a given contact map. ORCA and GRAFENE count more advanced graphlet substructures including graphlet orbits (which consider the relative node identity within the graphlet).

We also evaluated against two other methods that admit learned structure-based representations: DeepFRI and the Kipf and Welling GAE. Each method consists of training an autoencoder on contact maps and extracting average-pooled representations from one of the hidden layers in the inference mode. DeepFRI is a CNN autoencoder, whereas the GAE is a graph autoencoder. Both models are trained to minimize the binary cross-entropy of the original contact map and its reconstruction.

Of the five selected structure-based methods, four were permutation invariant; the exception was DeepFRI, which considers the canonical sequence ordering and treats the input matrix as an image. In addition, the manual crafted feature vectors do not scale well with graph density and hence cannot be evaluated for larger angstrom thresholds.

Evaluation metrics shown in Fig. 2b include cluster-adjusted mutual information and triplet-scoring AUPR. Each benchmark was applied to the top five most represented categories of each of the four CATH tiers separately. For cluster-adjusted mutual information, we applied spectral clustering using five clusters to the input feature vectors and calculated the adjusted mutual information between the cluster assignments and the actual label assignments. For triplet-scoring AUPR, we chose triplets in which two of the three shared the same label assignment, whereas the third was drawn from a different category. We constructed a balanced classification problem by considering the same-label pairs as the positive class and the same number of differently labeled pairs as the negative class. We used the cosine similarity among the selected positive and negative pairs as a classification prediction and calculated the AUPR.

Supplementary Tables 3 and 4 show the results of our comparison of TM-Vec with several methods on the CATHS20 benchmark and ProtTucker benchmarks. The commands used to run FoldSeek, HHBlits, MMseqs2 and Diamond are included in the TM-Vec software repository.

### Reporting summary

Further information on research design is available in the Nature Portfolio Reporting Summary linked to this article.

## Online content

Any methods, additional references, Nature Portfolio reporting summaries, source data, extended data, supplementary information, acknowledgements, peer review information; details of author contributions and competing interests; and statements of data and code availability are available at 10.1038/s41587-023-01917-2.

### Supplementary information

Supplementary Information Supplementary Figs. 1–9 and Tables 1–6.

Reporting Summary

## Data Availability

All the protein sequences and structures used in this study for training and evaluation are publicly available. CATH domain sequences and structures are publicly available at http://www.cathdb.info/ (ref. ^37^). SWISS-MODEL sequences and structures are available at https://swissmodel.expasy.org/ (ref. ^38^). Our evaluation included several different datasets. Malidup can be found at http://prodata.swmed.edu/malidup/ (ref. ^43^); Malisam at http://prodata.swmed.edu/malisam/ (ref. ^44^); the MIP at https://zenodo.org/record/6611431 (ref. ^48^); and the Bagel dataset at http://bagel.molgenrug.nl (ref. ^45^). DeepBLAST and TM-Vec are trained on protein sequences as well as TM-align outputs. Training and test datasets used for DeepBLAST can be found in the following repository: https://zenodo.org/record/7731163 (ref. ^83^); and training datasets for the different TM-Vec models can be found at https://zenodo.org/record/8038377 (ref. ^84^). Source code and data for all of the TM-Vec data visualizations are provided on Zenodo at https://zenodo.org/record/8021495 (ref. ^85^). Source code for all of the DeepBLAST data visualizations are provided at 10.5281/zenodo.7731163 (ref. ^86^).

## Code availability

TM-Vec can be found on GitHub at https://github.com/tymor22/tm-vec and on Zenodo at 10.5281/zenodo.8021495 (ref. ^85^). DeepBLAST can be found on GitHub at https://github.com/flatironinstitute/deepblast and on Zenodo at 10.5281/zenodo.8021480 (ref. ^86^). The structure visualizations presented in Figs. 1 and 3 were created using PyMOL v.2.4.0 (ref. ^87^). Our TM-Vec data visualizations were created using RStudio (v.2022.12.0+353) (ref. ^88^) and tidyverse (v.1.3.2) (ref. ^89^). Detailed installation instructions for installing both packages together or separately can be found at https://github.com/flatironinstitute/deepblast/wiki/Installation (ref. ^86^).
