# Supplementary material for: Protein remote homology detection and structural alignment using deep learning
Source: Nat Biotechnol. 2023 Sep 7;42(6):975–85. doi: 10.1038/s41587-023-01917-2 (PMC11180608; doi:10.1038/s41587-023-01917-2)
Supplement: Supplementary file 1 — Supplementary Figs. 1–9 and Tables 1–6. [file 41587_2023_1917_MOESM1_ESM.pdf]

nature biotechnology

Article

https://doi.org/10.1038/s41587-023-01917-2

Protein remote homology detection and structural alignment using deep learning

In the format provided by the authors and unedited

Supplemental Figures

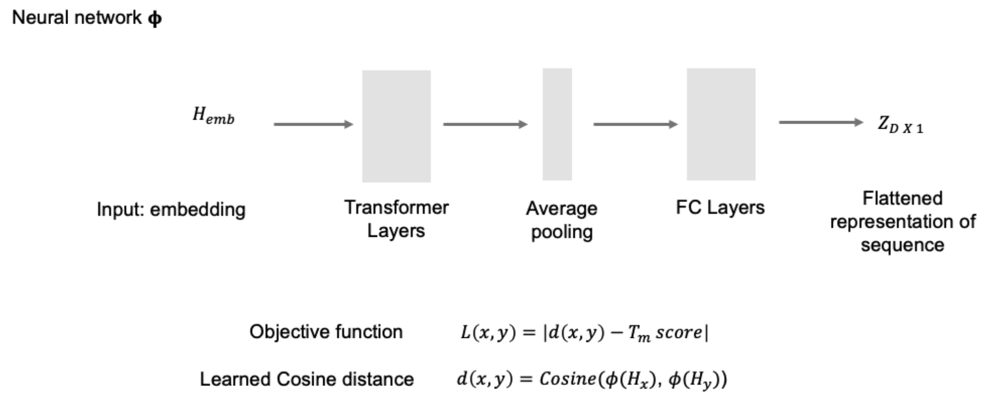

Figure S1: Overview of TM-Vec neural network architecture. The function φ takes in residue embeddings and produces a flattened vector representation for each protein. φ is composed of several transformer encoder layers, followed by average pooling, dropout, and fully connected layers. At the final step, we calculate the cosine distance between the vector representations of each protein in the pair, and our training objective is to minimize the L1 distance between the cosine similarity of the pairs vector representations, z, and their TM-score.

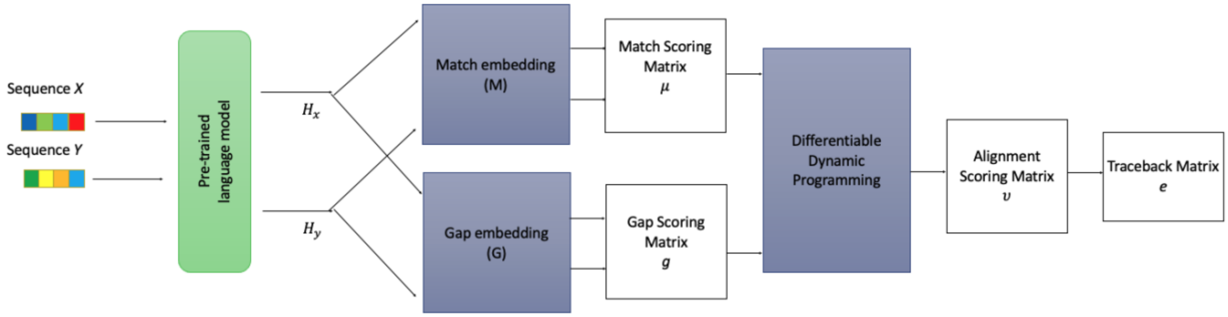

Figure S2: Overview of aligner pipeline. Proteins X and Y are fed into the pretrained protein language model, ProtTrans [35], to obtain embeddings H_X and H_Y. These residue-level embeddings are then propagated through the match embeddings (M) and gap embeddings (G) in order to obtain the match scores μ and the gap scores g. The match and gap scores are used to evaluate the differentiable dynamic programming algorithm and generate a predicted alignment traceback.

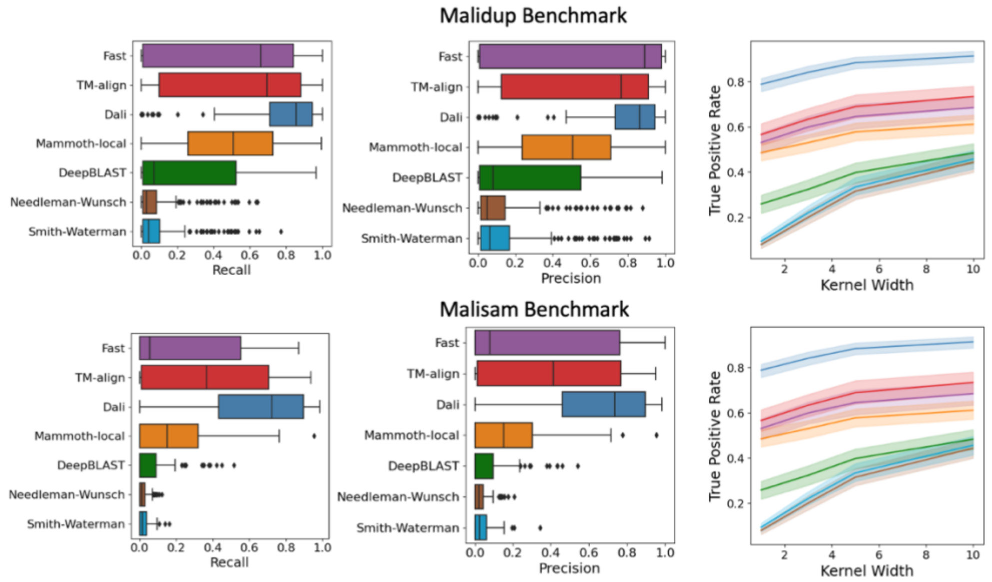

Figure S3: Precision and Recall metrics for each alignment on Malidup and Malisam benchmarks. We evaluated multiple methodologies (Fast, TM-align, Dali, Mammoth, DeepBLAST, Needleman-Wunsch and Smith-Waterman) on 234 proteins from the Malidup benchmark in addition to 129 proteins from the Malisam benchmark. The true positive rate was evaluated within window. Bounds of the boxplots denote 25% and 75% percentiles, the center is the mean and the whiskers are denoted by the 1.5 IQR. Data in the line plots are presented as mean values with 95% confidence intervals.

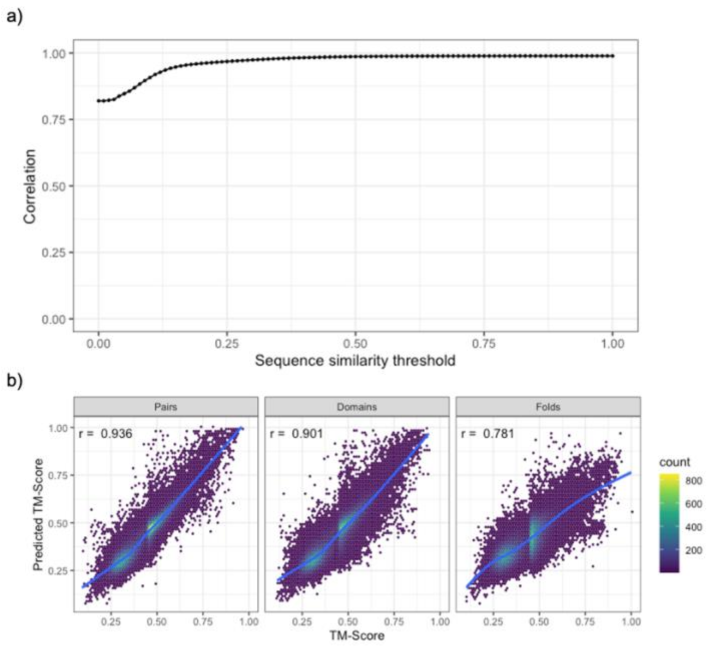

Figure S4: a) SWISS-MODEL cumulative Pearson correlations between the known TM-scores and predicted TM-scores for 1.01 million pairs of sequences at different sequence identity thresholds. The correlation coefficient at a particular value represents the correlation coefficient for SWISS-MODEL test pairs below that sequence identity threshold. b) CATH S40 predicted TM-scores versus ground truth TM-scores for 681K proteins across 3 different test datasets (Pairs, Domains, Folds). Contour plots show the density of points, and we also show the trend line for the relationship and Pearson correlation. The trend line is presented as mean values estimated with a LOESS fit with 95% confidence intervals.

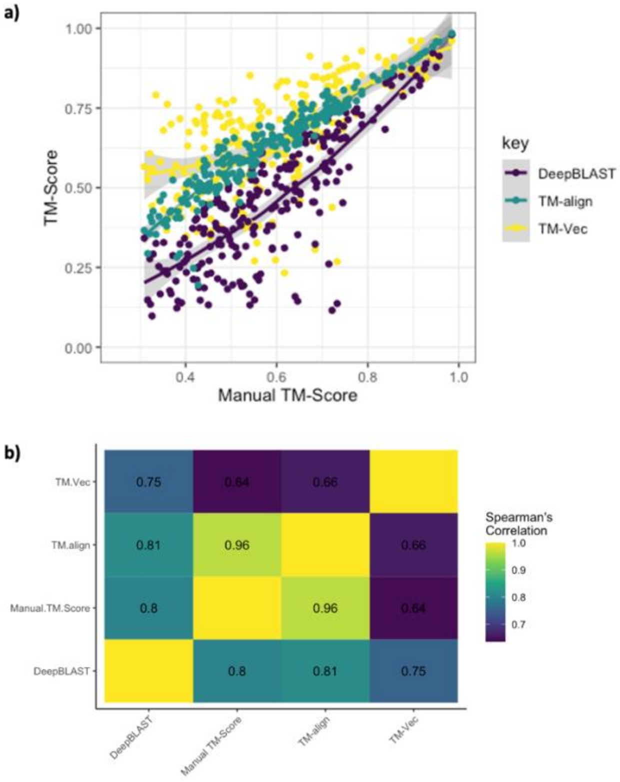

Figure S5: a) Comparison of TM-Vec, DeepBLAST, and TM-align on the Malidup benchmark. The Y-axis represents the predicted TM-score, and the X-axis represents the TM-score from a manually curated alignment. Data are presented as mean values estimated with a LOESS fit with 95% confidence intervals. b) Spearman's rank correlations between TM-Vec, DeepBLAST, TM-align, and the manually curated alignments on the Malidup benchmark.

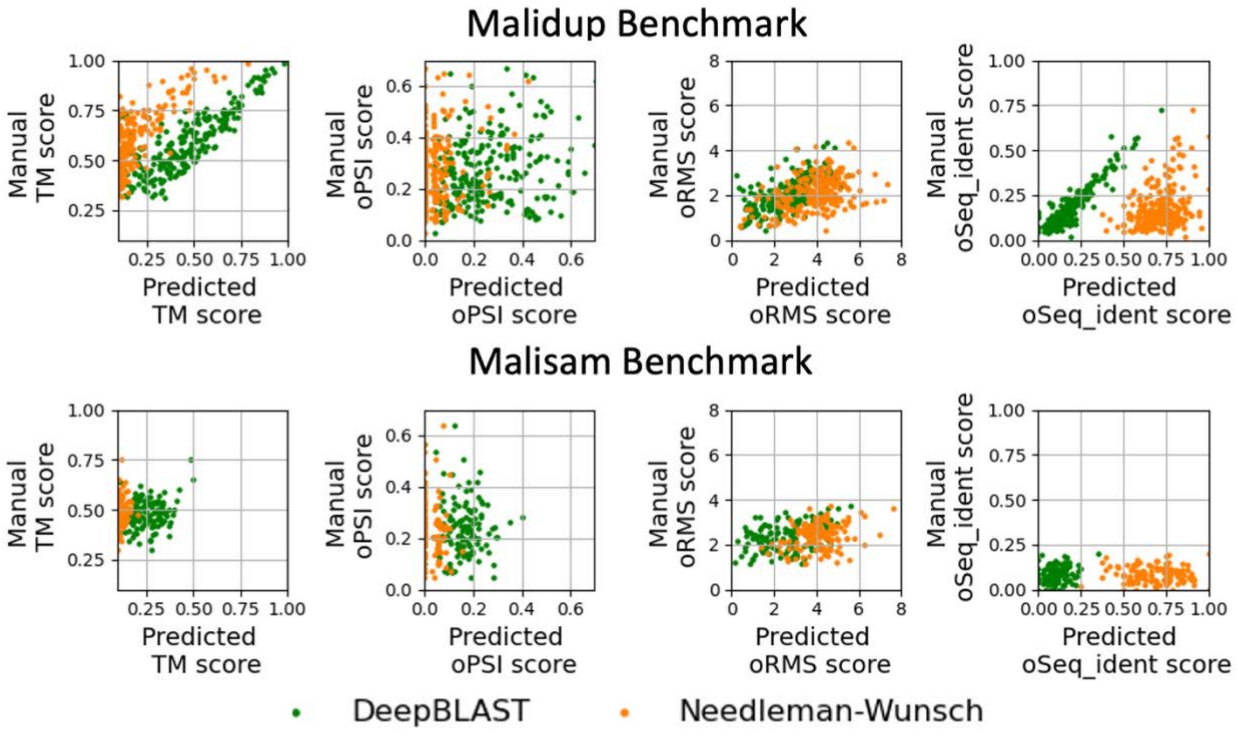

Figure S6: Comparison of DeepBLAST and Needleman-Wunsch on the Malidsam and Malidup benchmark. The points represent structural alignment metrics on the manually curated alignments, whereas the solid lines represent the structural alignment metrics of the corresponding DeepBLAST and Needleman-Wunsch alignments. TM-score measures the superposition agreement between the two aligned protein structures. The oPSI metric measures the fraction aligned residues relative to the smaller protein on the aligned residues predicted to strongly superimposed by the alignment method. The oRMS metric measures the root mean standard deviation of the atomic positions on the aligned residues predicted to strongly superimposed by the alignment method. The oSeq identity score measures the fraction of identical sequence measured over the subset of the sequence alignment that was also aligned structurally by method.

a)

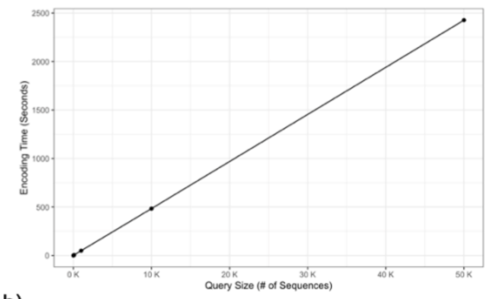

c)

Query Size	Database Size	Blast	TM-Vec	Diamond
10	1,000	9	0.6	0.521
100	1,000	16	5	0.512
1,000	1,000	65	50	0.597
10	10,000	10	0.7	0.573
100	10,000	25	5.2	0.644
1,000	10,000	131	50.2	0.775
10	100,000	14	0.7	1.234
100	100,000	63	5.3	1.395
1,000	100,000	475	51	1.743

b)

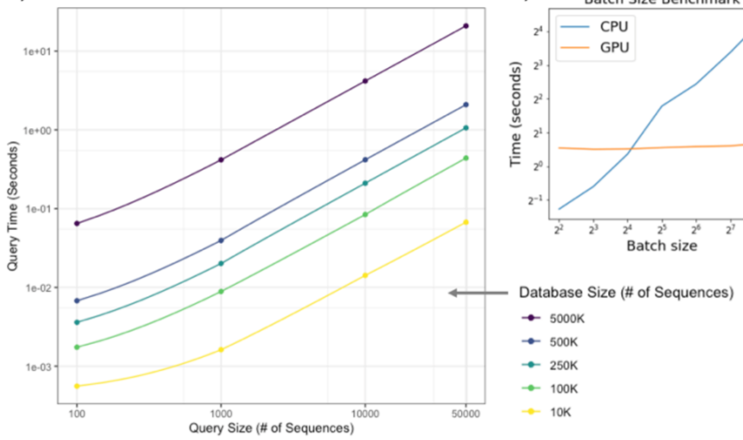

d)

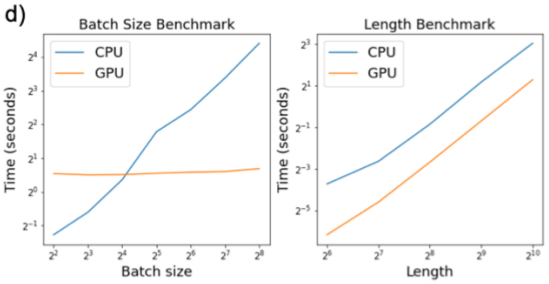

Figure S7: a) We evaluated how long it takes to encode query sequences using TM-Vec without parallelization on 1 GPU. The query size is on the x axis, and the encoding time in seconds is on the y axis. Encoding queries is linear in time. b) We evaluated how long it takes to search an indexed TM-Vec database and return the nearest neighbors once the queries have been encoded. The query size is on the x axis (number of sequences) and the query time is on the y axis (number of seconds) for databases of different sizes ranging from 10K sequences to 5M sequences. Search time is trivial relative to the time it takes to encode queries, and the size of the database does not materially impact speed. c) Search and alignment speed for TM-Vec is compared against BLAST, and DIAMOND for different query and database sizes. d) CPU vs GPU Differentiable Needleman-Wunsch benchmarks. The batch-size benchmark was run with randomized proteins of length 800 and the length benchmark was run with a fixed batch size of 64.

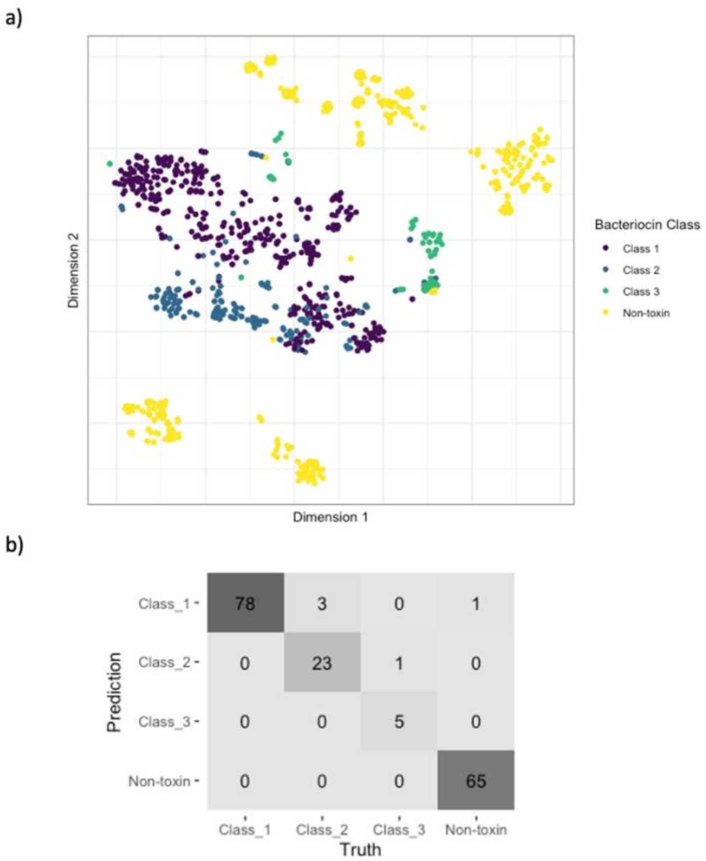

Figure S8: a) T-sne visualization of embeddings for both bacteriocins and non-toxin proteins. Clearly there is a separation of bacteriocins and non-bacteriocins. b) Confusion matrix for predicting the bacteriocin status/class for a held out test set of proteins. 85% of data was used to train a k-nearest neighbors classifier with k equal to 3 neighbors. The overall precision and recall on the held out test set were 0.98 and 0.93, respectively.

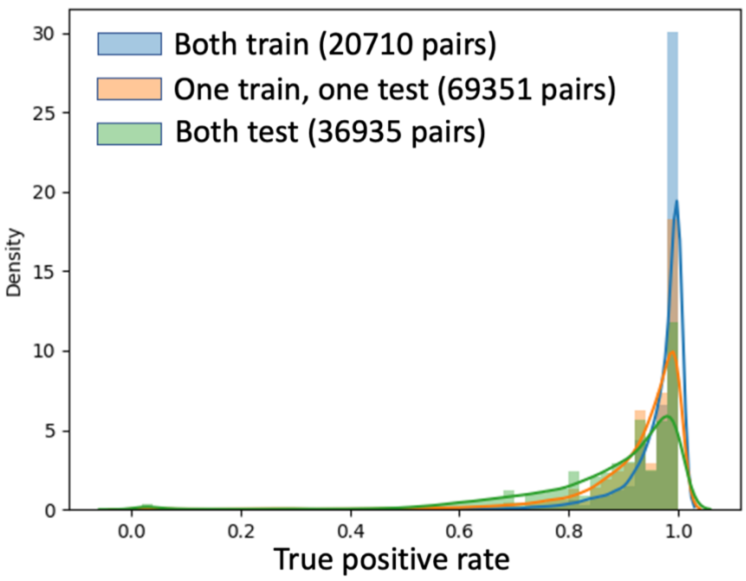

Figure S9: Distribution of DeepBLAST true positive rates across heldout alignment datasets.

Supplemental Tables

	Predicted TM>0.5	Predicted TM<0.5
Known TM>0.5	8,342<br>(true positives)	10<br>(false negatives)
Known TM<0.5	6,892<br>(false positives)	168,368<br>(true negatives)

Table S1: Predicting TM-scores for novel folds discovered by the Microbiome Immunity project (MIP). There was a Pearson correlation of .786 between TM-Vec's TM-score predictions and the known TM-scores for pairs of proteins from new, undiscovered folds discovered by the MIP project. This correlation is nearly identical to the correlation of 0.78 that we got predicting on a test dataset of left out folds from CATH.

Dataset	Tier	Top 1	Top 3	Top 5	Top 10
CATH S100	Class	98.9	99.3	99.5	99.6
	Architecture	98.6	99.2	99.4	99.6
	Topology	97.7	98.2	98.4	98.6
	Superfamily	96.9	97.3	97.4	97.7
CATH S40	Class	94.7	97.4	98.2	98.9
	Architecture	92.8	96.3	97.5	98.5
	Topology	88.1	90.8	91.9	93.4
	Superfamily	83	85.5	86.4	87.7

Table S2: TM-Vec search accuracy on CATH datasets across multiple tiers with varying sequence similarity. Across every tier of CATH, and for two CATH datasets, CATH S40 (clusters thresholded at 40% sequence similarity) and CATH S100 (thresholded at 100% sequence similarity), we show the classification accuracy of the nearest neighbors returned by TM-Vec. For example, the Top 1 accuracy for Topology in CATH S100 is 97.7%, which means that 97.7% of the time, the nearest neighbor returned using TM-Vec is in the same fold (topology level in CATH) as the query domain's fold. As another example, the Top 3 column for Topology indicates the percentage of the time that one of the top 3 nearest neighbors returned by TM-Vec shares the same fold as the query domain's fold.

	Class	Architecture	Topology	Homology
TM-Vec	88.50%	80.40%	70.60%	81%
ProtTucker (EAT)	88%	77%	68%	78%
FoldSeek	77.20%	72.50%	59%	77%
MMseqs2	53.40%	32.90%	21.10%	24.70%

Table S3: Classification across the CATH hierarchy on the ProtTucker/EAT evaluation dataset. This evaluation dataset consists of 219 domains that do not have an HVAL > 0 with any of the lookup or training dataset domains. CATH annotations are transferred from the lookup dataset to the query dataset. TM-Vec performance is based on the top returned nearest neighbor. TM-Vec retrieves the query domain's homology 81% of the time, compared to ProtTucker's (EAT) 78%, and FoldSeek's 77%.

	CATH ID (Homology)
TM-Vec (CATH)	88%
FoldSeek	85%
TM-Vec (SwissProt)	71%
ProtTucker (EAT)	71%
HHBlits	49%
MMSeq2 (E value of 1000)	36%
MMSeq2 (E value < .01)	27%
Diamond	26%

Table S4: Homology search accuracy on CATH S20. For different structure and sequence methods, homology classification accuracy on CATH S20 is compared (where CATH S20 is the lookup database and every domain is queried, and cannot match with itself). FoldSeek searches over the CATH S20 structures, while the other methods use the CATH S20 sequences. For the embedding-based methods (TM-Vec and ProtTucker), the homology classification comes from the nearest neighbor in embedding space. The TM-Vec CATH model is trained on the same domains as ProtTucker, and classifies domains with 88% accuracy compared to ProtTucker's 71%. A TM-Vec model trained on SWISS-MODEL chains performs similarly to ProtTucker at 71%. FoldSeek achieves 85% accuracy, while HHBlits has 49% accuracy.

	Maximum protein length	Top 1	Top 5	Top 10	Top 20	Top 50	Top 100
Single and multiple-domain proteins	600 residues	91.50%	94.50%	95.20%	95.80%	96.50%	97.00%
	1000 residues	92.10%	95.00%	95.70%	96.30%	96.90%	97.40%
Only multiple-domain proteins	600 residues	86.20%	92.90%	94.10%	94.90%	95.60%	96.00%
	1000 residues	82.60%	90.80%	92.40%	93.50%	94.60%	95.20%

Table S5: TM-Vec search sensitivity on the DIAMOND benchmark. TM-Vec search sensitivity on the DIAMOND benchmark, consisting of a UniRef50 lookup database of single and multiple-domain proteins and a query dataset of NCBI nr proteins. Every protein in the query and lookup databases is annotated with SCOP family annotations for its predicted domains. Sensitivity is reported as the percentage of retrieved nearest neighbor lookup proteins with the same family annotations as the queried proteins'. TM-Vec's performance is broken down by its performance on query/lookup proteins up to 600 residues long (1.36 million queries) as well as query/lookup proteins up to 1000 residues long (1.56 million queries) . TM-Vec's performance is also broken down on all queries versus only multiple-domain queries. For all query proteins, TM-Vec's first returned result shares the same family annotations as the query proteins' annotations 92.1% of the time. As another example, the Top 10 column indicates the percentage of the time that one of the top 10 nearest neighbors returned by TM-Vec shares the same family as the query proteins'; this is 95.7% for all query proteins up to 1000 residues long, and 92.4% for all multiple-domain proteins up to 1000 residues long.

Number of Queries	Number of Nearest Neighbors	Time (seconds) for 1M Lookup DB	Time (seconds) for 10M Lookup DB
100	1.00E+01	0	0.01
100	1.00E+02	0.01	0.02
100	1.00E+03	0.02	0.03
100	1.00E+04	0.04	0.06
100	1.00E+05	0.1	0.31
100	1.00E+06	0.81	1.13

Table S6: TM-Vec performance as the number of nearest neighbors grows. We evaluated how long it takes to retrieve different numbers of nearest neighbors for 100 encoded query vectors using TM-Vec without parallelization on 1 GPU. The number of nearest neighbors retrieved ranged from 10 to 1M, and the size of the lookup database used in these experiments ranged from 1M to 10M vectors.
