# Supplementary material for: Protein remote homology detection and structural alignment using deep learning
Source: Nat Biotechnol. 2023 Sep 7;42(6):975–85. doi: 10.1038/s41587-023-01917-2 (PMC11180608; doi:10.1038/s41587-023-01917-2)
Supplement: Supplementary file 2 — Reporting Summary [file 41587_2023_1917_MOESM2_ESM.pdf]

nature portfolio

Corresponding author(s): Richard Bonneau

Last updated by author(s): July 16th 2023

Reporting Summary

Nature Portfolio wishes to improve the reproducibility of the work that we publish. This form provides structure for consistency and transparency in reporting. For further information on Nature Portfolio policies, see our Editorial Policies and the Editorial Policy Checklist.

Please do not complete any field with "not applicable" or n/a. Refer to the help text for what text to use if an item is not relevant to your study. For final submission: please carefully check your responses for accuracy; you will not be able to make changes later.

Statistics

For all statistical analyses, confirm that the following items are present in the figure legend, table legend, main text, or Methods section.

n/a	Confirmed	
☐	☑	The exact sample size (n) for each experimental group/condition, given as a discrete number and unit of measurement
☑	☐	A statement on whether measurements were taken from distinct samples or whether the same sample was measured repeatedly
☐	☑	The statistical test(s) used AND whether they are one- or two-sided<br>Only common tests should be described solely by name; describe more complex techniques in the Methods section.
☑	☐	A description of all covariates tested
☑	☐	A description of any assumptions or corrections, such as tests of normality and adjustment for multiple comparisons
☐	☑	A full description of the statistical parameters including central tendency (e.g. means) or other basic estimates (e.g. regression coefficient) AND variation (e.g. standard deviation) or associated estimates of uncertainty (e.g. confidence intervals)
☑	☐	For null hypothesis testing, the test statistic (e.g. F, t, r) with confidence intervals, effect sizes, degrees of freedom and P value noted Give P values as exact values whenever suitable.
☑	☐	For Bayesian analysis, information on the choice of priors and Markov chain Monte Carlo settings
☑	☐	For hierarchical and complex designs, identification of the appropriate level for tests and full reporting of outcomes
☐	☑	Estimates of effect sizes (e.g. Cohen's d, Pearson's r), indicating how they were calculated

Our web collection on statistics for biologists contains articles on many of the points above.

Software and code

Policy information about availability of computer code

Data analysis

TM-align v.20190822 (https://zhanggroup.org//TM-align/) was used for computing TM-scores, sequence identities, and structure alignments for pairs of proteins and domains. This data was used for training both TM-Vec and DeepBLAST.

Data collection

TM-Vec can be found at https://github.com/tymor22/tm-vec. DeepBLAST can be found at https://github.com/flatironinstitute/deepblast. The versions of imports used by TM-Vec and DeepBLAST are included in their respective software repositories. For TM-Vec: https://github.com/tymor22/tm-vec/blob/master/setup.py, and for DeepBLAST: https://github.com/flatironinstitute/deepblast/blob/master/setup.py.

Detailed installation instructions for installing both packages together or separately can be found here: https://github.com/flatironinstitute/deepblast/wiki/Installation.Structure visualizations were created in Pymol v.2.4.0 (https://github.com/schrodinger/pymol-open-source). For our TM-Vec data visualizations, we used R version 4.2.2 (2022-10-31), with RStudio Version 2022.12.0+353, tidyverse 1.3.2.

All of the structural alignments analyzed were provided from the original benchmarks: MALIDUP:https://pubmed.ncbi.nlm.nih.gov/17932926/, MALISAM: https://pubmed.ncbi.nlm.nih.gov/17855399/). Structural encoding comparison was done using this repository: https://github.com/djberenberg/structure-encoding.

The commands used to run FoldSeek, HHBlits, MMseqs2, and Diamond are included in the TM-Vec software repository. We also compared TM-Vec with 3 structure predictions methods for this benchmark, including AlphaFold2, ESMFold, and OmegaFold. The versions used were DIAMOND v2.0.14, MMseqs2 Release 14-7e284, Foldseek 3-915ef7d, HH-suite3 (3.3.0), ProtTucker (release date of DBs: 16.11.2021), ESMFold: ESM-2 Public Release v1.0.3, OmegaFold: OmegaFold v1.1.0, and AlphaFold2: AlphaFold v2.3.1.

For manuscripts utilizing custom algorithms or software that are central to the research but not yet described in published literature, software must be made available to editors and reviewers. We strongly encourage code deposition in a community repository (e.g. GitHub). See the Nature Portfolio guidelines for submitting code & software for further information.

Data

Policy information about availability of data

All manuscripts must include a data availability statement. This statement should provide the following information, where applicable:

- Accession codes, unique identifiers, or web links for publicly available datasets
- A description of any restrictions on data availability
- For clinical datasets or third party data, please ensure that the statement adheres to our policy

Training and test datasets used for DeepBLAST can be found in the following repository: https://zenodo.org/record/4117030; and training datasets for the different TM-Vec models can be found here: https://zenodo.org/record/8038377.

All of the protein sequences and structures used in this study for training and evaluation are publicly available. CATH domain sequences and structures are publicly available here: http://www.cathdb.info/. SWISS-MODEL sequences and structures are available here: https://swissmodel.expasy.org/.

Our evaluation included several different datasets. Malidup can be found here: http://prodata.swmed.edu/malidup/; Malisam can be found here: http://prodata.swmed.edu/malisam/; the Microbiome Immunity Project data can be found here: https://zenodo.org/record/6611431; and the Bagel dataset can be found here: http://bagel.molgenrug.nl.

Source code and data for all of the TM-Vec data visualizations are provided on Zenodo at https://zenodo.org/record/8021495. Source code for all of the DeepBLAST data visualizations are provided here: https://zenodo.org/record/4117030.

Research involving human participants, their data, or biological material

Policy information about studies with human participants or human data. See also policy information about sex, gender (identity/presentation), and sexual orientation and race, ethnicity and racism.

Reporting on sex and gender	NA
Reporting on race, ethnicity, or other socially relevant groupings	NA
Population characteristics	NA
Recruitment	NA
Ethics oversight	NA

Note that full information on the approval of the study protocol must also be provided in the manuscript.

Field-specific reporting

Please select the one below that is the best fit for your research. If you are not sure, read the appropriate sections before making your selection.

☑ Life sciences ☐ Behavioural & social sciences ☐ Ecological, evolutionary & environmental sciences

For a reference copy of the document with all sections, see nature.com/documents/nr-reporting-summary-flat.pdf

Life sciences study design

Sample size	No sample size calculation was performed; we trained and tested on all of our available data, including millions of protein pairs and thousands of proteins. Information on sample sizes for different training experiments and tests are provided in the Online Methods, Results and Supplement.
Data exclusions	Information on data exclusions is provided in the Online Methods. All of our data exclusions are pre-established and have to do with training and testing models that handle proteins of different lengths. We did not train or test on proteins longer than 1000 residues long.
Replication	All of our code and training data are publicly available to replicate our findings.
Randomization	We used randomization during the training/validation/testing of our models. Information on randomization is provided in the Online Methods and the Supplement.
Blinding	Used during testing/evaluation - information on blinding is provided in the Online Methods and the Supplement.

All studies must disclose on these points even when the disclosure is negative.

Behavioural & social sciences study design

Study description	
Research sample	
Sampling strategy	
Data collection	
Timing	
Data exclusions	
Non-participation	
Randomization	

All studies must disclose on these points even when the disclosure is negative.

Ecological, evolutionary & environmental sciences study design

Study description	
Research sample	
Sampling strategy	
Data collection	
Timing and spatial scale	
Data exclusions	
Reproducibility	
Randomization	
Blinding	

Did the study involve field work? ☐ Yes ☐ No

All studies must disclose on these points even when the disclosure is negative.

Field work, collection and transport

Field conditions

Location

Access & import/export

Disturbance

Reporting for specific materials, systems and methods

We require information from authors about some types of materials, experimental systems and methods used in many studies. Here, indicate whether each material, system or method listed is relevant to your study. If you are not sure if a list item applies to your research, read the appropriate section before selecting a response.

Materials & experimental systems

n/a	Involved in the study
☑	☐ Antibodies
☑	☐ Eukaryotic cell lines
☑	☐ Palaeontology and archaeology
☑	☐ Animals and other organisms
☑	☐ Clinical data
☑	☐ Dual use research of concern
☑	☐ Plants

Methods

n/a	Involved in the study
☑	☐ ChIP-seq
☑	☐ Flow cytometry
☑	☐ MRI-based neuroimaging

Antibodies

Antibodies used

Validation

Eukaryotic cell lines

Policy information about cell lines and Sex and Gender in Research

Cell line source(s)

Authentication

Mycoplasma contamination

Commonly misidentified lines
(See ICLAC register)

Palaeontology and Archaeology

Specimen provenance

Specimen deposition

Dating methods

☐ Tick this box to confirm that the raw and calibrated dates are available in the paper or in Supplementary Information.

Ethics oversight

Note that full information on the approval of the study protocol must also be provided in the manuscript.

Animals and other research organisms

Policy information about studies involving animals; ARRIVE guidelines recommended for reporting animal research, and Sex and Gender in Research

Laboratory animals	
Wild animals	
Reporting on sex	
Field-collected samples	
Ethics oversight	

Note that full information on the approval of the study protocol must also be provided in the manuscript.

Clinical data

Policy information about clinical studies

All manuscripts should comply with the ICMJE guidelines for publication of clinical research and a completed CONSORT checklist must be included with all submissions.

Clinical trial registration	
Study protocol	
Data collection	
Outcomes	

Dual use research of concern

Policy information about dual use research of concern

Hazards

Could the accidental, deliberate or reckless misuse of agents or technologies generated in the work, or the application of information presented in the manuscript, pose a threat to:

No	Yes	
☐	☐	Public health
☐	☐	National security
☐	☐	Crops and/or livestock
☐	☐	Ecosystems Any other
☐	☐	significant area

Experiments of concern

Does the work involve any of these experiments of concern:

No	Yes	
☐	☐	Demonstrate how to render a vaccine ineffective
☐	☐	Confer resistance to therapeutically useful antibiotics or antiviral agents
☐	☐	Enhance the virulence of a pathogen or render a nonpathogen virulent
☐	☐	Increase transmissibility of a pathogen
☐	☐	Alter the host range of a pathogen
☐	☐	Enable evasion of diagnostic/detection modalities
☐	☐	Enable the weaponization of a biological agent or toxin
☐	☐	Any other potentially harmful combination of experiments and agents

Plants

Seed stocks

Novel plant genotypes

Authentication

ChIP-seq

Data deposition

☐ Confirm that both raw and final processed data have been deposited in a public database such as GEO.

☐ Confirm that you have deposited or provided access to graph files (e.g. BED files) for the called peaks.

Data access links
May remain private before publication.

Files in database submission

Genome browser session
(e.g. UCSC)

Methodology

Replicates

Sequencing depth

Antibodies

Peak calling parameters

Data quality

Software

Flow Cytometry

Plots

Confirm that:

☐ The axis labels state the marker and fluorochrome used (e.g. CD4-FITC).

☐ The axis scales are clearly visible. Include numbers along axes only for bottom left plot of group (a 'group' is an analysis of identical markers).

☐ All plots are contour plots with outliers or pseudocolor plots.

☐ A numerical value for number of cells or percentage (with statistics) is provided.

Methodology

Sample preparation

Instrument

Software

Cell population abundance

Gating strategy

☐ Tick this box to confirm that a figure exemplifying the gating strategy is provided in the Supplementary Information.

Magnetic resonance imaging

Design type

Design specifications

Behavioral performance measures

Imaging type(s)

Field strength

Sequence & imaging parameters

Area of acquisition

Diffusion MRI ☐ Used ☐ Not used

Experimental design

Preprocessing

Preprocessing software

Normalization

Normalization template

Noise and artifact removal

Volume censoring

Statistical modeling & inference

Model type and settings

Effect(s) tested

Specify type of analysis: ☐ Whole brain ☐ ROI-based ☐ Both

Statistic type for inference

(See Eklund et al. 2016)

Correction

Models & analysis

n/a	Involved in the study
☒	☐ Functional and/or effective connectivity
☒	☐ Graph analysis
☒	☐ Multivariate modeling or predictive analysis

Functional and/or effective connectivity

Graph analysis

Multivariate modeling and predictive analysis

This checklist template is licensed under a Creative Commons Attribution 4.0 International License, which permits use, sharing, adaptation, distribution and reproduction in any medium or format, as long as you give appropriate credit to the original author(s) and the source, provide a link to the Creative Commons license, and indicate if changes were made. The images or other third party material in this article are included in the article's Creative Commons license, unless indicated otherwise in a credit line to the material. If material is not included in the article's Creative Commons license and your intended use is not permitted by statutory regulation or exceeds the permitted use, you will need to obtain permission directly from the copyright holder. To view a copy of this license, visit http://creativecommons.org/licenses/by/4.0/
